# Comprehensive molecular characterization of gastric cancer patients from phase II second-line ramucirumab plus paclitaxel therapy trial

**DOI:** 10.1186/s13073-021-00826-w

**Published:** 2021-01-25

**Authors:** Seung Tae Kim, Jason K. Sa, Sung Yong Oh, Kyung Kim, Jung Yong Hong, Won Ki Kang, Kyoung-Mee Kim, Jeeyun Lee

**Affiliations:** 1 Division of Hematology-Oncology, Department of Medicine, Samsung Medical Center, Sungkyunkwan University School of Medicine, 81 Irwon-ro, Gangnam-gu, Seoul, 06351 Republic of Korea; 2 BK21 Graduate Program, Department of Biomedical Sciences, Korea University College of Medicine, Seoul, Republic of Korea; 3 Dong-A University School of Medicine, Busan, Republic of Korea; 4 Department of Pathology and Translational Genomics, Samsung Medical Center, Sungkyunkwan University School of Medicine, Seoul, Republic of Korea

**Keywords:** TCGA molecular subtype, Angiogenesis signature, Ramucirumab, Gastric cancer

## Abstract

**Background:**

Gastric cancer (GC) is a heterogenous disease consisted of several subtypes with distinct molecular traits. The clinical implication of molecular classification has been limited especially in association with treatment efficacy of ramucirumab or various targeted agents.

**Methods:**

We conducted a prospective non-randomized phase II single-arm trial of ramucirumab plus paclitaxel as second-line chemotherapy in 62 patients with metastatic GC who failed to respond to first-line fluoropyrimidine plus platinum treatment. For integrative molecular characterization, all patients underwent pre-ramucirumab treatment tissue biopsy for whole-exome/whole-transcriptome sequencing to categorize patients based on molecular subtypes. We also systematically performed integrative analysis, combining genomic, transcriptomic, and clinical features, to identify potential molecular predictors of sensitivity and resistance to ramucirumab treatment.

**Results:**

Sixty-two patients were enrolled in this study between May 2016 and October 2017. Survival follow-up in all patients was completed as of the date of cut-off on January 2, 2019. No patient attained complete response (CR), while 22 patients achieved confirmed partial response (PR), resulting in a response rate (RR) of 35.5% (95% CI, 23.6–47.4). According to TCGA molecular classification, there were 30 GS, 18 CIN, 3 EBV, and 0 MSI tumors. The RR was 33% in GS (10/30), 33% in CIN (6/18), and 100% in EBV-positive GC patients with significant statistical difference for EBV(+) against EBV(−) tumors (*P* = 0.016; chi-squared test). Moreover, responsive patients were marked by activation of angiogenesis, VEGF, and TCR-associated pathways, while non-responder patients demonstrated enrichments of sonic hedgehog signaling pathway and metabolism activity. Integrative multi-layer data analysis further identified molecular determinants, including EBV status, and somatic mutation in *GNAQ* to ramucirumab activity.

**Conclusions:**

Prospective molecular characterization identified a subset of GC patients with distinct clinical response to ramucirumab therapy, and our results demonstrate the feasibility of personalized therapeutic opportunities in gastric cancer.

**Trial registration:**

The study was registered on ClinicalTrial.gov (NCT02628951) on June 12, 2015.

**Supplementary Information:**

The online version contains supplementary material available at 10.1186/s13073-021-00826-w.

## Background

In 2012, gastric cancer (GC) was diagnosed in approximately 951,000 patients, which led to the death of 723,000 worldwide [1]. Despite great improvements in anti-cancer therapy for solid tumors, the prognosis of metastatic GC remains dismal [2]. Ramucirumab is a recombinant human IgG1-neutralizing monoclonal antibody (mAb), specific for ectodomain of vascular endothelial growth factor receptor (VEGFR-2). In 2014, the RAINBOW trial (phase III, paclitaxel versus paclitaxel/ramucirumab as second-line chemotherapy) demonstrated a significantly prolonged survival in the combination group (median of 9.6 months) compared to the paclitaxel-alone group (7.4 months), along with a higher response rate (paclitaxel/ramucirumab, 28% versus paclitaxel-alone, 16%) [3]. As a result, the paclitaxel/ramucirumab combination has become the second-line standard of care for treatment of metastatic GC worldwide.

GC consists of molecularly heterogenous subgroups with different prognoses, following curative surgery or chemotherapy [4–7]. The Cancer Genome Atlas (TCGA) Research Network and The Asian Cancer Research Group (ACRG) have identified four distinct molecular classification of GC based on molecular signatures [4, 5]. The four major genomic subtypes of GC categorized by TCGA include tumors positive for (1) Epstein-Barr virus (EBV), (2) microsatellite instability (MSI), (3) chromosomal instability (CIN), and (4) genome stability (GS), while the ACRG proposed (1) MSI, (2) microsatellite stability (MSS)/epithelial-mesenchymal transition (EMT), (3) MSS/TP53(+), and (4) MSS/TP53(−) with distinct survival outcomes. These molecular subtypes with accompanying unique genomic alterations have provided a basis for future patient-centered stratification and application of targeted therapies. In 2018, we have demonstrated significant anti-tumor activities of pembrolizumab (anti-PD1 antibody) in MSI and EBV(+) GCs [8]. However, the optimal subset of GC patients that may benefit the most from anti-angiogenesis inhibitors, such as ramucirumab, has not been clearly defined. Interestingly, enrichments of angiogenesis- and cell-adhesion pathways including integrin and syndecan-1 were prevalent in a subset of GC patients [4, 9], suggesting potential clinical benefits of ramucirumab. Recurrent genomic amplification of *VEFGA* was highly recognized in tumors with CIN, followed by GS in the TCGA dataset. Moreover, several stromal and/or angiogenesis-related gene signatures had been previously developed in GC as well [10]. We hypothesized that these unique transcriptional angiogenic signatures may provide complimentary information to accompany the pre-existing TCGA/ACRG subtypes, when predicting clinical response to anti-angiogenic therapy such as treatment of ramucirumab.

In this study, we have conducted a prospective phase II trial of ramucirumab plus paclitaxel as second-line (2L) treatment in 62 patients with metastatic GC who failed to respond to first-line fluoropyrimidine/platinum (with or without trastuzumab). All patients were subjected to pre-ramucirumab treatment tissue biopsy with whole-exome and/or whole-transcriptome sequencing to categorize patients based on molecular classification. We also overlaid the molecular subtypes with gene expression signatures that are specific for tumor-associated angiogenesis. Lastly, we have conducted machine-learning-based integrative molecular analysis to identify specific genomic and/or transcriptomic correlates of clinical response to ramucirumab therapy. The primary objective of this study was to combine direct assessment of clinical outcomes with comprehensive molecular characterization, in hopes of facilitating the identification of molecular biomarker that could aid in the design of effective treatments for ramucirumab and paclitaxel in GC patients.

## Methods

### Study procedure

This prospective open-label phase II trial was designed as a single-arm phase II study at an academic cancer center. Ramucirumab (8 mg/kg) was intravenously administered on days 1 and 15, and paclitaxel (80 mg/m^2^) was intravenously administered on days 1, 8, and 15. This regime was repeated every 4 weeks. The treatment continued until disease progression was documented, or unacceptable toxicity, or patients’ refusal was reported. Tumor responses were evaluated after every two cycles, according to RECIST 1.1 criteria [11]. Toxicities were graded based on the National Cancer Institute Common Terminology Criteria for Adverse Events 4.0. We are reporting on the final analysis.

### Eligibility criteria

Patients enrolled in this study had measurable and histologically or cytologically confirmed metastatic and/or recurrent gastric adenocarcinoma. The trial was conducted in accordance with the Declaration of Helsinki and the Guidelines for Good Clinical Practice (ClinicalTrial.gov identifier: NCT# 02628951). The trial protocol (Additional file 1) was approved by the Institutional Review Board of Samsung Medical Center (Seoul, Korea), and all patients provided written informed consent before the enrolment. To be eligible for participating in this study, the key inclusion criteria were as follows (detailed eligibility criteria are provided in clinicaltrials.gov NCT#02628951): (1) the patient has histologically or cytologically confirmed gastric carcinoma, including gastric adenocarcinoma or GEJ adenocarcinoma; (2) metastatic disease or locally recurrent, unresectable disease; (3) measurable or evaluable disease as determined by standard computed tomography (CT) or magnetic resonance imaging (MRI) imaging; (4) disease progression during treatment or within 4 months after the last dose of first-line therapy for metastatic disease; (5) not amenable to potentially curative resection; (6) ≥ 18 years of age; (7) resolution to grade ≤ 1 (or to grade ≤ 2 in the case of neuropathy) by the National Cancer Institute Common Terminology Criteria for Adverse Events (NCI- CTCAE), version 4.03, of all clinically significant toxic effects of prior chemotherapy, surgery, radiotherapy, or hormonal therapy (with the exception of alopecia); (8) Eastern Cooperative Oncology Group performance status (ECOG PS) score of 0 or 1; (9) adequate hepatic function as defined by a total bilirubin ≤ 1.5 mg/dL (25.65 μmol/L), and aspartate transaminase (AST) and alanine transaminase (ALT) ≤ 3.0 times the upper limit of normal (ULN; or 5.0 times the ULN in the setting of liver metastases); (10) patient does not have cirrhosis at a level of Child-Pugh B (or worse) or cirrhosis (any degree) and a history of hepatic encephalopathy or clinically meaningful ascites resulting from cirrhosis—clinically meaningful ascites is defined as ascites from cirrhosis requiring diuretics or paracentesis; (11) adequate renal function as defined by a serum creatinine ≤ 1.5 times the ULN, or creatinine clearance (measured via 24-h urine collection) ≥ 40 mL/min (that is, if serum creatinine is > 1.5 times the ULN, a 24-h urine collection to calculate creatinine clearance must be performed); (12) urinary protein is ≤ 1+ on dipstick or routine urinalysis; (13) adequate hematologic function, as evidenced by an absolute neutrophil count (ANC) ≥ 1000/μL, hemoglobin ≥ 9 g/dL (5.58 mmol/L), and platelets ≥ 100,000/μL; (14) adequate coagulation function as defined by international normalized ratio (INR) ≤ 1.5 and a partial thromboplastin time (PTT) ≤ 5 s above the ULN (unless receiving anticoagulation therapy); (15) has received prior anthracycline therapy as part of his or her first-line regimen, the patient is able to engage in ordinary physical activity without significant fatigue or dyspnea (equivalent to New York Heart Association Class I function); (16) because the teratogenicity of ramucirumab is not known, the patient, if sexually active, must be postmenopausal, surgically sterile, or using effective contraception (hormonal or barrier methods); (17) female patients of childbearing potential must have a negative serum pregnancy test within 7 days prior to enrollment; (18) is able to provide informed written consent; and (19) has feasible biopsy site.

The exclusion criteria were as follows: (1) The patient has documented and/or symptomatic brain or leptomeningeal metastases; (2) experienced any grade 3 to 4 GI bleeding within 3 months prior to enrollment; (3) experienced any arterial thromboembolic events, indicating but not limited to myocardial infarction, transient ischemic attack, cerebrovascular accident, or unstable angina, within 6 months prior to enrollment; (4) has an ongoing or active infection, symptomatic congestive heart failure, unstable angina pectoris, symptomatic or poorly controlled cardiac arrhythmia, uncontrolled thrombotic or hemorrhagic disorder, or any other serious uncontrolled medical disorders in the opinion of the treating physician; (5) has ongoing or active psychiatric illness or social situation that would limit compliance with treatment; (6) has uncontrolled or poorly controlled hypertension (> 160 mmHg systolic or > 100 mmHg diastolic for > 4 weeks) despite standard medical management; (7) has a serious or nonhealing wound, ulcer, or bone fracture within 28 days prior to enrollment; (8) received chemotherapy, radiotherapy, immunotherapy, or targeted therapy for gastric cancer within 2 weeks prior to enrollment; (9) received any investigational therapy within 30 days prior to enrollment; (10) has undergone major surgery within 28 days prior to enrollment, or subcutaneous venous access device placement within 7 days prior to enrollment; (11) received prior therapy with an agent that directly inhibits VEGF (including bevacizumab), or VEGF receptor 2 activity, or any anti-angiogenic agent; (12) is receiving chronic antiplatelet therapy, including aspirin, nonsteroidal anti-inflammatory drugs (NSAIDS; including ibuprofen, naproxen, and others), dipyridamole or clopidogrel, or similar agents—once-daily aspirin use (maximum dose 325 mg/day) is permitted; (13) has elective or planned major surgery to be performed during the course of the clinical trial; (14) has a known allergy to any of the treatment components; (15) is pregnant or breastfeeding; (16) is known to be positive for infection with the human immunodeficiency virus (HIV); (17) has known alcohol or drug dependency; (18) has a concurrent active malignancy other than adequately treated non-melanomatous skin cancer, other noninvasive carcinomas, or in situ neoplasm; (19) has a known hypersensitivity to ramucirumab or any of the excipients; and (20) may not have received more than 1 prior therapy in the metastatic setting.

### Tumor sample collection

Tumor tissues were obtained anytime between days 1 and 42 prior to the initiation of ramucirumab treatment. If tumor content was estimated to be ≥ 40% after pathological review, tumor DNA and RNA were extracted from freshly obtained tissues using a QIAamp Mini Kit (Qiagen, Hilden, Germany) according to the manufacturer’s instructions. In case of DNA, we used RNaseA (cat. #19101; Qiagen). We determined concentrations and absorbance ratios, OD_260_/OD_280_ and OD_260_/OD_230_, with an ND1000 spectrophotometer (NanoDrop Technologies, Thermo-Fisher Scientific, MA, USA) and quantified DNA/RNA using a Qubit fluorometer (Life Technologies, CA, USA).

### MSI status determination and EBV in situ hybridization

MSI status of tumor tissue was determined in formalin-fixed paraffin-embedded tissue sections for MLH1 (antibody: ES05 clone; 1:100 dilution; Novocastra, UK) and MSH2 (clone G219-1129; 1: 500 dilution; CELL Marque; Rocklin, CA, USA) by IHC and PCR analysis of five markers with mononucleotide repeats (*BAT-25*, *BAT-26*, *NR-21*, *NR-24*, and *NR-27*), as previously described. Briefly, each sense primer was end-labeled with FAM, HEX, or NED. Pentaplex PCR was performed, and the PCR products were run on an Applied Biosystems PRISM 3130 automated genetic analyzer. Allele sizes were estimated using GeneScan 2.1 software (Applied Biosystems, Foster City, CA, USA). Samples with allelic size variations in more than two microsatellites were considered MSI-H. EBV status was determined by EBER in situ hybridization using standard protocols [12].

### Whole-exome sequencing for tumor tissue

For the generation of standard exome capture libraries, we used the Agilent SureSelect Target Enrichment protocol for Illumina paired-end sequencing library (ver. B.3, June 2015), together with 200 ng input FFPE DNA. In all cases, the SureSelect Human All Exon V5 probe set was used. Quantification of DNA and DNA quality was conducted by PicoGreen [13] and NanoDrop, respectively [14]. Fragmentation of genomic DNA was performed using adaptive focused acoustic technology (AFA; Covaris). The fragmented DNA was repaired, an “A” was ligated to the 3′ end, and Agilent adapters were ligated to the fragments. Once ligation had been assessed, the adapter-ligated product was PCR amplified. The final purified product was then quantified using qPCR according to the qPCR Quantification Protocol Guide and assessed using the Caliper High Sensitivity DNA LabChip Kit (PerkinElmer). For exome capture, 250 ng of DNA library was mixed with hybridization buffers, blocking mixes, RNase block, and 5 μL of SureSelect all exon capture library, according to the standard Agilent SureSelect Target Enrichment protocol. Hybridization to capture baits was performed at 65 °C, using heated lid option of thermocycler at 105 °C for 24 h on a PCR machine. The captured DNA was then amplified. The final purified product was eventually quantified using qPCR according to the qPCR Quantification Protocol Guide and assessed using the TapeStation RNA ScreenTape (Agilent). Finally, we performed sequencing using the HiSeq™ 2500 platform (Illumina, San Diego, USA).

### Identification of somatic mutation

The adapter sequences were removed by cutadapt (v1.9.1) and FastQC software was used for quality control of the FASTQ files. Sequenced reads were aligned to the human reference genome (hg19) using Burrows-Wheeler Aligner (BWA, v0.7.17) [14]. Poorly mapped reads with mapping quality (MAPQ) below 20 were removed using Samtools (v1.3.1). After pre-processing of exome sequencing data, using Genome Analysis Toolkit (local reorganization, duplicate marking, indel realignment, and basic recalibration), Samtools (v1.8), and Picard (part of the GATK package, v4.0.3.0), somatic mutations, including single nucleotide variations (SNVs) and small insertions and deletions (Indels), were identified using MuTect2 [15]. SNVs with total read counts < 20 were also removed. Somatic mutations were annotated with Annovar (v2018Apr16) [16]. Copy number variations were detected using Mutect2 with default parameters. Variant calls were further analyzed using the COSMIC database, dbSNP build 142, and amino acid change information.

### Mutation signature analysis

Mutational signature analysis was performed using *deconstructSigs* (v1.8.0) [17] in R that selects the combination of known mutational signatures (v2), which can account for the observed mutational profile in each sample. Exome regions were defined by Agilent SureSelect v6-post target region. Only somatic mutations in exome regions were considered, and tri-nucleotide counts were normalized by the number of times each tri-nucleotide occurred in the exome region.

### RNA sequencing

Total RNA concentration was estimated by Quant-IT RiboGreen (Invitrogen). To determine the DV200 (% of RNA fragments > 200 bp) value, samples were run on the TapeStation RNA ScreenTape (Agilent). Overall, 100 ng of total RNA was subjected to sequencing library construction using a TruSeq RNA Access library prep kit (Illumina, San Diego, CA, USA) according to the manufacturer’s protocol. Briefly, the total RNA was first fragmented into small pieces using divalent cations under elevated temperature. The cleaved RNA fragments were copied into first-strand cDNA using SuperScript II reverse transcriptase (Invitrogen, #18064014) and random primers. This was followed by second-strand cDNA synthesis using DNA polymerase I, RNase H, and dUTP. These cDNA fragments were subjected to an end-repair process, addition of a single “A” base, and subsequently, ligation of the adapters. The products are then purified and enriched with PCR to create the cDNA library. All libraries were normalized and six were pooled into a single hybridization/capture reaction. Pooled libraries were incubated with a cocktail of biotinylated oligos, corresponding to coding regions of the genome. Targeted library molecules were captured via hybridized biotinylated oligo probes using streptavidin-conjugated beads. After two rounds of hybridization/capture reactions, the enriched library molecules were subjected to a second round of PCR amplification. The captured libraries were quantified using KAPA Library Quantification kits for Illumina Sequencing platforms according to the qPCR Quantification Protocol Guide (KAPA BIOSYSTEMS, #KK4854) and assessed using the TapeStation D1000 ScreenTape (Agilent Technologies, # 5067-5582). Indexed libraries were then submitted to an Illumina HiSeq2500 (Illumina, Inc., San Diego, CA, USA), and paired-end (2 × 100 bp) sequencing was performed by Macrogen Incorporated.

### Gene expression calling

RNA sequence reads were trimmed using trimmomatic (v 0.36) [18]. Trimmed reads were mapped to the reference human genome (GRCh37, hg19) using HISAT2 (v2.1.0) [19]. On the basis of Ensembl gene annotation, transcripts were assembled using Cufflinks (v2.2.1) [20]. Subsequently, gene expression levels of transcripts were calculated using Cuffdiff (part of the Cufflinks package), on the basis of Fragments per Kilobase per Million (FPKM) reads method.

### Gene expression signature analysis

FPKM reads were converted to the log10 scale and then quantile normalized. RNA signatures were calculated as the average gene expression in the signature. We used the previously defined signature [8] along with the stromal-based signature.

### Single-sample Gene Set Enrichment Analysis

Single-sample Gene Set Enrichment Analysis (ssGSEA) is an adaptation of the Gene Set Enrichment Analysis and generates an enrichment score of a given gene set for an individual sample. Each enrichment score represents the degree in which the genes in a given gene set are either up- or downregulated in a single sample. For ramucirumab-resistant signature analysis, we generated an input file that consists of normalized gene expression data across samples and an enrichment score was computed based on a list of genes that were highly expressed in ramucirumab non-responders via differentially expressed gene analysis.

### Gene signature analysis for EMT, proliferation, and p53

We calculated the EMT, proliferation, and p53 gene expression signature scores using the average of log intensity (also known as the geometric average) of expression of the corresponding genes in the signature [5].

### Elastic-net regression model-based integrative analysis

We selected 48 gastric cancer patients with available RNA-seq, Whole-Exome seq, and clinical response to ramucirumab. The input variables for the elastic-net regression model-based analysis consisted of gene expression profiles, genomic alterations including mutations, and copy number alterations. We trained the standard elastic-net regression using the *glmnet* R package by combining input features and comparing to individual clinical response, including tumor reduction rate, overall survival, or progression-free survival. Afterwards, we employed bootstrapping strategy for 100 times to extract reliable and robust candidate features. During each bootstrapping step, we randomly selected 80% of the tumors for feature extraction. For each feature, the time of its appearance out of 100 bootstrapping and its average weight were used as the final assessment.

### Sample size and statistical analysis

A maximum of 61 patients was planned to be recruited to this single-arm phase II trial. The primary endpoint of this trial is overall response (OR = PR + CR). Wilke et al. [3] observed 16% of OR rate (ORR) from a combination therapy called RAINBOW. We will not be interested in the experimental therapy of this trial if its ORR is P0 = 15% or lower and highly interested if its ORR is P1 = 30% or higher. A maximum of *n* = 58 eligible patients (61 accounting for 5% of ineligibility) will be treated through the following 2-stage design. Stage 1: n1 = 30 patients will be treated by the experimental therapy, and the trial will be stopped by rejecting the experimental therapy if 4 or fewer of them respond. Otherwise, we will proceed to stage 2. Stage 2: An additional 28 patients will be treated, and we will reject the study therapy if 13 or fewer of the cumulative 58 patients respond. Otherwise, the experimental therapy will be accepted for further investigation. This 2-stage design has a one-sided alpha of 5% for P0 = 15% and a power of 86% for P1 = 30%. To analyze the response rate according to molecular subtypes identified through ACRG/TCGA effort, we performed an integrative genomic analysis to identify predictive markers (i.e., angiogenesis signatures) for treatment response. All statistical analyses were performed using R3.4.0. Data was imported into R, plotted, computed, and *P* values automatically added for significance levels, using “ggpubr” and visualized using “ggplot2.”

## Results

### Clinical manifestation of gastric cancer patients

**Fig. 1:**
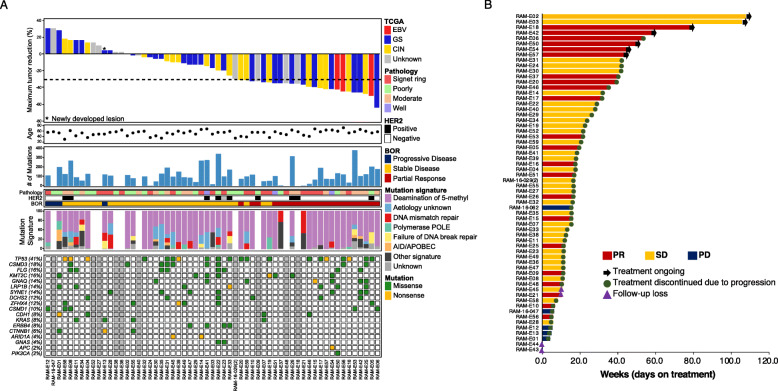
Genomic landscape of GC patients in response to ramucirumab. **a** Genomic landscape of gastric cancer patients with waterfall plot of response to ramucirumab (top panel). The *y*-axis represents the percentage of maximum tumor reduction assessed according to RECIST 1.1 criteria. The lower dotted line represents tumor reduction of 30%, as per RECIST, representing partial response (PR). The bar graphs are colored based on TCGA molecular classification. The first middle panel represents age distribution, the second middle panel depicts overall mutational burden (non-synonymous mutation), the third middle panel shows histopathological classification, the fourth panel demonstrates HER2 expression level, the fifth panel demonstrates objective response rate to ramucirumab and paclitaxel response, and the last middle panel represents mutational signatures. The bottom panel demonstrates mutational landscape. **b** Swimmer plot. Each lane represents a single patient’s data. The *x*-axis represents the duration of ramucirumab therapy for each patient

Sixty-two patients were enrolled in this study between May 26, 2016, and October 31, 2017 (Table 1). All survival follow-up in all patients were completed as of the date of cut-off on January 2, 2019. The median age of the patients was 59 years (range, 27–81 years), and the majority were men (45/62, 72.6%) (Fig. 1a). Six (9.7%) patients had tumors located within the cardia or gastroesophageal junction. Two-thirds (47 of 62) of the patients had more than two sites of metastatic involvement. Three patients (4.8%) were confirmed as EBV(+), and ten patients (16.1%) demonstrated positive expression for HER2. All patients exhibited microsatellite stability and underwent pre-treatment biopsy (47 stomach/primary tumor, 8 liver, 4 peritoneal, 1 lung, 1 distant lymph node, and 1 soft tissue mass) prior to the enrollment. In total, 51 tumor specimens were subjected to whole-exome sequencing (tumors with either insufficient tumor volume or DNA quality were excluded); 48 cases were of sufficiently high quality for whole-transcriptome sequencing (CONSORT, Additional file 2). The observed toxicity profile for the treatment was as per expectation and is provided in Table 2 .

**Table 1:** Baseline characteristics

	Total (***n*** = 62)
Age (years)	59 (27–81)
Sex	
Male	45 (72.6%)
Female	17 (27.4%)
Race	
Asian	62
ECOG performance status	
0	9 (14.5%)
1	53 (85.5%)
Primary tumor site	
Cardia	6 (9.7%)
Body	31 (50.0%)
Antrum	25 (40.3%)
Tumor grade	
Well-differentiated adeno	3 (4.8%)
Moderately differentiated	17 (27.4%)
Poorly differentiated	22 (35.5%)
Signet ring cell type	20 (32.3%)
Disease status	
Recurrent after surgery	5 (8.1%)
Metastatic at diagnosis	57 (91.9%)
Number of metastatic sites	
1 organ	16 (25.8%)
≥ 2 organs	46 (74.2%)
HER2 positivity	10 (16.1%)
EBV positivity	3 (4.8%)
MSS	62 (100.0%)

**Table 2:** Toxicity profile

	Grades 1–2	Grade 3	Grade 4	Grade 5
Non-hematological adverse events				
Fatigue	15 (24.2%)	0	0	0
Neuropathy	23 (37.1%)	0	0	0
Anorexia	22 (35.5%)	0	0	0
Alopecia	41 (66.1%)	0	0	0
Diarrhea	6 (9.7%)	0	0	0
Epistaxis	5 (8.1%)	0	0	0
Vomiting	5 (8.1%)	1 (1.6%)	0	0
Peripheral edema	6 (9.7%)	1 (1.6%)	0	0
Hypertension	8 (12.9%)	5 (8.1%)	0	0
Constipation	5 (8.1%)	0	0	0
Dyspnea	7 (11.3%)	0	0	0
Weight decreased	5 (8.1%)	0	0	0
Ascites	3 (4.8%)	0	0	0
Myalgia	6 (9.7%)	0	0	0
Nausea	9 (14.5%)	0	0	0
Rash	8 (12.9%)	1 (1.6%)	0	0
Mucositis	11 (17.7%)	0	0	0
Back pain	4 (6.5%)	0	0	0
Cough	4 (6.5%)	0	0	0
Hematologic adverse events				
Neuropenia	13 (21.0%)	16 (25.8%)	5 (8.1%)	0
Febrile neutropenia	0	1 (1.6%)	0	0
Anemia	18 (29.0%)	2 (3.2%)	0	0
Thrombocytopenia	2 (3.2%)	0	0	0

### Molecular characterization of GC patients in response to ramucirumab treatment

The endpoint for treatment outcome analysis was determined as of January 2, 2019, where assessable clinical responses were available for 57 patients with a median follow-up at 30.2 months. In an intention-to-treat analysis cohort, there was no CR and 22 patients achieved confirmed PRs, resulting in an objective response rate (ORR) of 35.5% (95% CI, 23.6–47.4%) (Fig. 1a). Of the 22 patients who achieved PR, two experienced more than 50% reduction in tumor burden, as per Response Evaluation Criteria in Solid Tumors (RECIST) 1.1 (Fig. 1a). The duration of response spanned over 40 weeks in multiple patients (Fig. 1b).

We first evaluated the somatic mutation spectrum, focusing on mismatch repair (MMR) deficiency, homologous recombination repair deficiency, smoking, and landscape of mutational load etiology that can be inferred from the mutational signature analysis (Fig. 1a). Our cohort constituted no patient with MSI or a somatic hypermutator phenotype. For tumors with relatively high numbers of non-synonymous mutations (greater than 100 mutations), ORR was achieved at 38.5% (5 of 13), while low-mutational burden tumors (less than 100 mutations), demonstrated 47.2% (17 of 36) of ORR, supporting that overall somatic mutational burden does not confer clinical response to ramucirumab. Furthermore, histopathological features such as differentiated status did not correlate with ramucirumab response either. Interestingly, among molecular characteristics, including single nucleotide variations, copy number alterations, and protein expression level of HER2, somatic mutation in *GNAQ* was more commonly observed in patients with increased response to ramucirumab (Fig. 1a).

**Fig. 2:**
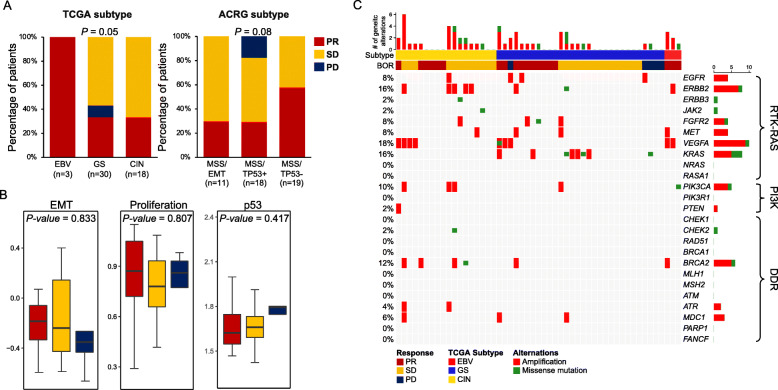
Ramucirumab response based on molecular classification. **a** Percentage of GC patients with clinical response based on TCGA (left panel) and ACRG (right panel) classification. **b** Box plots demonstrating pathway enrichment scores of each corresponding pathway. Box plots span from the first to third quantiles, and the whiskers represent the 1.5 interquartile range. **c** Genomic landscape of gastric patients focusing on RTK-RAS, PI3K, and DDR encoding genes. Genomic amplifications are highlighted in red and mutations are highlighted in green. Patients are ordered based on TCGA molecular classification. *P* values in **a** were derived from chi-squared tests, and the *P* values in **b** were derived from one-way ANOVA

Next, we sought to determine potential correlates of ramucirumab response based on genomic subtypes and associated molecular signatures based on previous TCGA and ACRG results. For the TCGA subtype, tumors were first classified as EBV, determined by EBER in situ hybridization and then by MSI status. Afterwards, the remaining tumors were categorized as either GS (genomically stable) or CIN (chromosomal instability) based on the degree of aneuploidy, determined by chromosomal-wide copy number alterations [4]. For ACRG classification, we employed both MSI/MSS and EMT signature activities to determine tumor classification [21, 22]. We have previously demonstrated that MSI and EMT signatures exhibited a mutually exclusive pattern and identified a subset of gastric tumors that were categorized as either MSI or MSS/EMT [5]. Within TCGA subtypes, EBV status, albeit low in number, demonstrated a significant sensitivity to ramucirumab, with confirmed response in all three patients (*P* = 0.05; chi-squared test) against EBV(−) patients. Conversely, both GS and CIN tumors constituted similar distribution levels in ORR (Fig. 2a). On the other hand, according to the ACRG molecular subtype, the responders were highly clustered in MSS/TP53 inactive status, compared to both MSS/EMT and MSS/TP53(+) (Fig. 2a). When we evaluated essential cellular processes that govern malignant phenotypic state of GCs, including epithelial-to-mesenchymal transition, cellular proliferation kinetics, and DNA damage and repair (p53), we did not discover any distinguishable attributes that contribute to ramucirumab response (Fig. 2b).

One of the intriguing observations was the presence of recurrent genomic amplification in the gene encoding ligand VEGFA. In the TCGA cohort, *VEGFA* amplification was specifically enriched in the CIN subtype, followed by GS in the order of magnitude. Additionally, recurrent focal amplifications of receptor tyrosine kinases (*EGFR*, *ERBB2*, *ERBB3*, and *MET*) or cell cycle mediators (*CCNE1*, *CCND1*, and *CDK6*) were previously suggested as potential therapeutic targets in GCs. Consistently, 18% of the patients harbored genomic amplification of *VEGFA*, 4, 3, and 2 in CIN, GS, and EBV subtypes, respectively (Fig. 2c). Furthermore, genomic aberrations in RTK encoding genes were prevalent in the CIN subtype. Unfortunately, despite its direct association, genomic alteration in *VEGFA* did not exhibit predictive potentiality to the clinical response of ramucirumab in this study (*P* = 0.2656), potentially due to the limited number of cases. Overall, we identified EBV status to be the potential predictive candidate for ramucirumab therapy in GC patients.

### Identification of molecular and transcriptional determinants that dictate the clinical response to ramucirumab

**Fig. 3:**
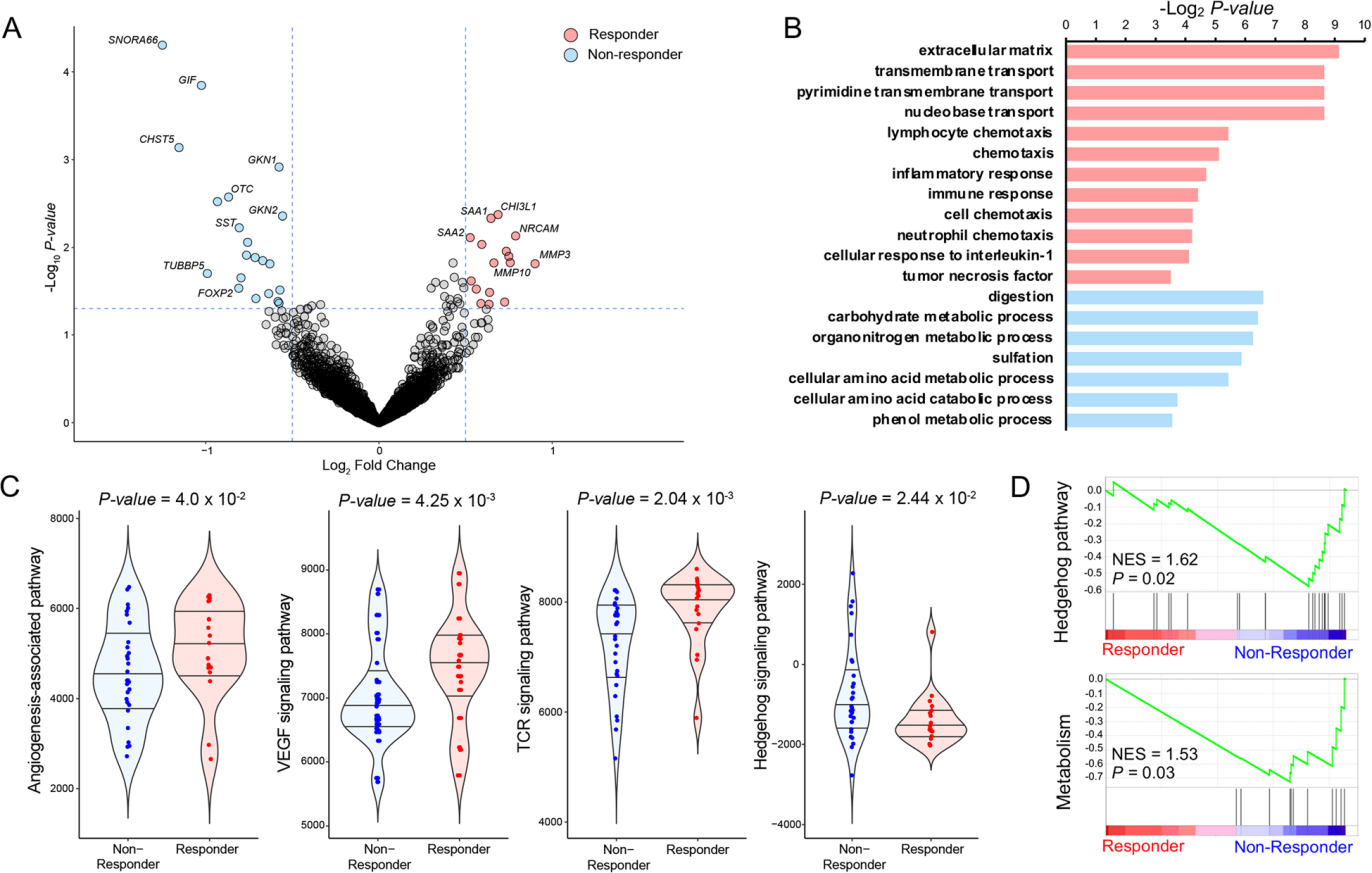
Transcriptome correlates of clinical response to ramucirumab. **a** Volcano plot representation of differentially expressed gene analysis between ramucirumab responders (patients who obtained partial response) and non-responders (patients who achieved stable or progressive disease). Genes with > 0.5 log_2_ fold change and < 0.05 *P* value are colored in red, and those with < − 0.5 log_2_ fold change and < 0.05 *P* value are colored in blue. **b** Gene Ontology (GO) analysis of differentially expressed genes from **a**. **c** Violin plot representations of the pathway enrichment scores via single-sample Gene Set Enrichment Analysis (ssGSEA). Horizontal lines within the violin plots represent 0.25, 0.50, and 0.75 quantiles. *P* values are calculated using two-sided Wilcoxon’s rank-sum test. **d** Gene Set Enrichment Analysis (GSEA) between ramucirumab-sensitive and resistance patients

Transcriptome analysis facilitates the identification of unique gene signature correlates for drug sensitivity [23–26]. To assess distinct transcriptional features that dictate the clinical response to ramucirumab in GC patients, we performed genome-wide differentially expressed gene analysis between ramucirumab responders and non-responders. Notably, responsive patients demonstrated high transcriptional levels in *CHI3L1*, *NRCAM*, and *MMP3* genes (Fig. 3a), which were associated with the extracellular matrix, transmembrane transport, chemotaxis, and immune response based on Gene Ontology analysis (Fig. 3b). Consistently, immune cell composition analysis revealed abundance of immune cell populations, including activated mast cells and CD4 memory T cells in the responder group (Additional file 3: Figure S1). Conversely, a set of transcriptomes that were enriched in the non-responder patients, termed “ramucirumab-resistant signature,” was associated with several biological mechanisms, including cellular metabolic and catabolic process. Furthermore, the ramucirumab-resistant signature demonstrated a significant association with gastric cancer prognosis, where patients with elevated expression of the ramucirumab-res signature portrayed worse clinical probabilities in TCGA cohort (Additional file 3: Figure S2). We further identified activation of angiogenesis, VEGF, NF-KB, and T cell receptor-associated pathways in the ramucirumab-responder group, whereas non-responder patients showed enrichments of metabolism activity and hedgehog signaling axis, suggesting potential therapeutic opportunities for hedgehog-mediated therapies (Fig. 3c, d and Additional file 3: Figure S3). Notably, cell migration and angiogenesis-associated pathways including Beta1/3, integrin, and syndecan-1 were highly enriched in the GS subtype based on TCGA molecular classification (Additional file 3: Figure S4).

**Fig. 4:**
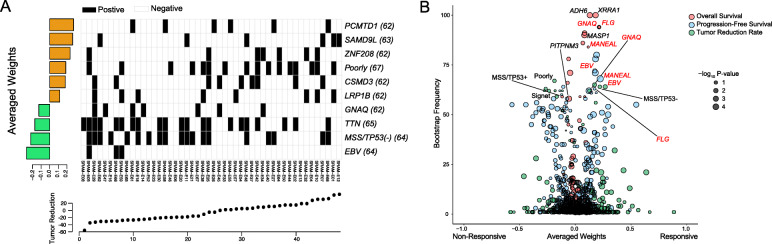
Identification of molecular determinants to ramucirumab response via integrative analysis. **a** Elastic-net regression results of transcriptome and genomic features that predict clinical response to objective response rate. The bottom scatter plots represent ramucirumab response based on tumor reduction rate. The upper heatmap shows the top extracted features in the model. The left bar graph shows the averaged weight of each predictive feature. The number of appearance from 100 bootstraps is indicated in parentheses. **b** Predictive features of ramucirumab response identified by the elastic-net regression model-based analysis are plotted based on their appearance frequency and effect size. Associations are colored in red for overall survival, blue for progression-free survival, and green for tumor reduction rate. The averaged weights for all the features that were extracted against tumor reduction rate were plotted inversely to show consistency in clinical prognosis. Node size is proportional to the single clinical-feature linear correlation

Assessment of clinical response to a targeted agent is a complex process that often depends on multiple variables. Therefore, we sought to identify potential genomic or transcriptional correlates of clinical response to ramucirumab via elastic-net regression model-based analysis, combining multiple layers of variables, including gene expression profiles, genomic features, and clinical information. As a result, we have discovered a group of molecular predictors against ramucirumab clinical responses, including tumor reduction rate, progression-free survival (PFS), and overall survival (OS). Consistent with previous observations, somatic mutation in *GNAQ* and EBV status were significantly associated with favorable prognosis in gastric cancer patients based on ORR, PFS, and OS outcomes (Fig. 4a, b, Additional file 3: Figure S5). Moreover, patients with MSS/TP53(−) status were more susceptible to ramucirumab treatment compared to their counterparts MSS/TP53(+) tumors. Collectively, our results provide therapeutically exploitable genomic and transcriptomic markers of drug sensitivity that may aid in the design of effective ramucirumab treatment in GC patients.

## Discussion

Our study constitutes the first comprehensive approach in characterizing molecular profiles of metastatic GC patients who received ramucirumab and paclitaxel as second-line treatment. All tumor specimens were procured immediately prior to ramucirumab treatment to account for any potential genetic drift due to selective pressure via therapeutic intervention. The overall response rate that was observed in this trial recapitulated the previous clinical outcomes from the RAINBOW trial (phase III trial) [3], and the MSI-H patient cohort was not enrolled in this study due to the proven efficacy of pembrolizumab in the MSI-H cohort [27].

GC is a heterogeneous disease, which can be subcategorized into distinct subtypes based on their transcriptional cellular state and accompanying unique genomic alterations and molecular signatures. This molecular-based classification system has emerged as an important concept in comprehending the biological behavior and genomic complexity of GC [4, 5, 28]. Its significance has been increasingly recognized owing to distinct clinical response to current treatment modalities, their diverse cellular originations, and differentiation hierarchies. While previous studies have shown great success in clinical application of ramucirumab in GC treatment, the potential predictor of clinical response to ramucirumab remains elusive. Toward this goal, we have performed integrative genomic and transcriptomic analysis of GC patients who received ramucirumab treatment, in hopes of facilitating more progressive course within the clinical framework. Through this approach, we have identified a subset of GC patients, specifically EBV(+) and MSS/TP53(−), with considerable targeted vulnerabilities to ramucirumab therapy. We have further identified a catalogue of transcriptomes that were specifically upregulated in non-responders, termed the “ramucirumab-resistant” signature, which significantly associated with unfavorable survival outcomes in TCGA gastric cancer patients. While ramucirumab responders constituted activation of several essential migratory processes, including angiogenesis, chemotaxis, and VEGF signals, enrichments of metabolism and sonic hedgehog cellular pathways were pertinent to non-responders. These results have led us to propose potential therapeutic strategy for the use of hedgehog targeting agents such as vismodegib to circumvent intrinsic resistance to VEGFR-mediated therapy. Moreover, we have identified molecular determinants, such as somatic mutation in *GNAQ* that dictate ramucirumab sensitivity. As gain of function mutations in G protein subunit α q (*GNAQ*) have been previously speculated to drive tumor malignancy in melanoma and gastric cancer via activation of mitogen-activated protein kinase (MAPK) pathway [29, 30], we propose that genetic alteration of *GNAQ* renders tumor cells susceptible to ramucirumab treatment, the underlying molecular mechanism of which warrants further investigation.

In conclusion, our results identified multiple molecular and transcriptional features related to clinical response of ramucirumab therapy in patients with GC and depicted alternative therapeutic avenue to overcome its intrinsic resistance. While ramucirumab did not pertain clinical improvements for all patients with GC, our study identified a subset of patients who may significantly benefit from such treatment, highlighting more personalized approach for refining patient stratification for ramucirumab therapy. On the contrary, our study does hold few limitations. First, we only enrolled patients with measurable lesions according to RECIST 1.1; thus, there was a discrepancy in the metastatic features between the study cohort and patients in clinics. Second, since all patients that were enrolled in our study received ramucirumab with paclitaxel, the biomarkers we identified could potentially reflect both drugs. While further clinical validation within a larger cohort is required, we provide a constructive groundwork for the implementation of individualized treatment in future clinical trials involving ramucirumab.

## Conclusions

Comprehensive genomic characterization of gastric tumors identified a subset of patients with distinct molecular profiles that respond to ramucirumab therapy, advocating clinical feasibility of precision medical treatment in gastric cancer.

## Supplementary Information

**Additional file 1.** Clinical trial study protocol.

**Additional file 2.** Clinical annotation of 61 gastric cancer patients that were enrolled in this study.

**Additional file 3: Figure S1.** Immune cell composition of gastric cancer patients based on clinical response to ramucirumab. **Figure S2.** TCGA gastric cancer patient survival analysis based on ramucirumab-resistant signature activities. **Figure S3.** Pathway enrichment analysis between ramucirumab responder and non-responder patients. **Figure S4.** A heatmap of angiogenesis-associated pathway analysis. **Figure S5.** Elastic net-regression model-based analysis.

## Data Availability

Due to the regulations of the institution, individual-level sequencing and detailed clinical data from this study cannot be uploaded to the public repository domain. Sequencing datasets that were used and/or analyzed in the current study as well as the study protocol and individual participant data that underlie the results reported in this article, after deidentification, will be shared on request to researchers who provide a methodologically sound proposal. It will be shared to achieve aims in the approved proposal. Requests for access to the data, including the data presented here, can be made to jyunlee@skku.edu. Requestors will be provided with information and assistance on how data can be accessed following submission of appropriate documentation that contains the requestor’s research proposal.
