# Supplementary material for: Comprehensive molecular characterization of gastric cancer patients from phase II second-line ramucirumab plus paclitaxel therapy trial
Source: Genome Med. 2021 Jan 25;13:11. doi: 10.1186/s13073-021-00826-w (PMC7836461; doi:10.1186/s13073-021-00826-w)
Supplement: Supplementary file 1 — Additional file 1. Clinical trial study protocol. [file 13073_2021_826_MOESM1_ESM.docx]

# Phase II ramucirumab/paclitaxel as second-line treatment in metastatic gastric or GEJ adenocarcinoma with integrative genomic analysis

### Number:

**Product Name:** Ramucirumab

**Indication:** second-line, gastric cancer

**Physicians:** Single center

**Sponsor:** Samsung Medical Center

**Sponsor Address:** Irwonro 81 , Kangnamgu, Seoul,

Korea

# SYNOPSIS

| **Sponsor:**  Samsung Medical Center |
| --- |
| **Name of Finished Product:**  Ramucirumab |
| **Name of Active Ingredient:**  Ramucirumab, a recombinant human immunoglobulin G, subclass 1 (IgG1) monoclonal antibody (MAb) targeted to the human vascular endothelial growth factor receptor-2 (VEGF Receptor 2) |
| **Title:**  **Phase II ramucirumab/paclitaxel as second-line treatment in metastatic gastric or GEJ adenocarcinoma with integrative genomic analysis** |
| **Protocol Number:** |
| **Primary Objective:**  To evaluate response rate of Ramucirumab in combination with paclitaxel per RECIST 1.1 |
| **Secondary Objectives:**  To analyze the response rate according to molecular subtypes identified through the ACRG effort, with a integrative genomic analysis to identify predictive markers (i.e. angiogenesis signatures) for treatment response |
| **Design:**  Single-arm, open-label protocol |

| **Population:**  Eligible patients (patients with advanced gastric adenocarcinoma who have progressed following 1 prior systemic therapy for metastatic disease) will be treated with ramucirumab and paclitaxel until such time as the patient is no longer benefiting from ramucirumab or is discontinued from protocol therapy for any reason. |
| --- |
| **Test Product, Dose, and Mode of Administration:**  Ramucirumab injection for intravenous (I.V.) use, supplied in single-use 500-mg/50-mL vials containing 10 mg/mL of product in histidine buffer, administered as an I.V. infusion after dilution at 8 mg/kg every 2 weeks in the absence of disease progression, toxicity requiring cessation, or withdrawal for any other reason. Paclitaxel will be administered at a dose of 80 mg/m2 on Days 1, 8 and 15 of a 28-day cycle in the absence of disease progression, toxicity requiring cessation, or withdrawal for any other reason. |
| **Duration of Treatment:**  Patients may be treated until there is evidence of progressive disease (PD), toxicity requiring cessation, or a decision is reached to cease ramucirumab therapy for any other reason. Patients wishing to continue ramucirumab therapy are advised to have evaluation of disease control every 6 weeks in order to ensure ongoing treatment with ramucirumab remains in their best interests. |
| **Safety Assessments:**  Serious adverse events, grade 3 or higher AEs, and AESIs of any grade will be the safety information collected; these events will be reported as specified in the protocol. |
| **Statistical Methods:**  A maximum of 61 patients will be recruited to this single-arm phase II trial. The primary endpoint of this trial is overall response (OR=PR+CR). Wilke et al. (27) observed 16% of OR rate (ORR ) from a combination therapy called RAINBOW. We will not be interested in the experimental therapy of this trial if its ORR is P0=15% or lower and highly interested if its ORR is P1=30% or higher. A maximum of n=58 eligible patients (61 accounting for 5% of ineligibility) will be treated through the following 2-stage design.  Stage 1: n1=30 patients will be treated by the experimental therapy, and the trial will be stopped by rejecting the |
| experimental therapy if 4 or fewer of them respond. Otherwise, we will proceed to Stage 2.  Stage 2: An additional 28 patients will be treated, and we will reject the study therapy if 13 or fewer of the |
| cumulative 58 patients respond. Otherwise, the experimental therapy will be accepted for further investigation. This 2-stage design has a one-sided alpha of 5% for P0=15% and a power of 86% for P1=30%.  **Statistical Analysis Plan**  To analyze the response rate according to molecular subtypes identified through ACRG/TCGA effort, we performed integrative genomic analysis to identify predictive markers (i.e. angiogensis signatures) for treatment response. All statistical analyses were performed using R3.4.0. Data was imported into R, plotted, computed, and P-values automatically added for significance levels, using “ggpubr” and visualized using “ggplot2”. |

# TABLE OF CONTENTS

- 1. [SYNOPSIS 2](#_TOC_250072)
  2. [TABLE OF CONTENTS 4](#_TOC_250071)
  3. [LIST OF ABBREVIATIONS AND DEFINITIONS OF TERMS 7](#_TOC_250070)
  4. [INTRODUCTION 10](#_TOC_250069)
     1. [Gastric Cancer 10](#_TOC_250068)
        1. [Background 10](#_TOC_250067)
        2. [First-Line Therapy 10](#_TOC_250066)
        3. [Second-Line Therapy 11](#_TOC_250065)
        4. [Table 1. Adverse Events According to Grade in REGARD 13](#_TOC_250064)
        5. Table 2. Treatment-Emergent Adverse Events Occurring in ≥20% of Patients and 14
        6. [Table 3. Adverse Events of Special Interest in RAINBOW 15](#_TOC_250063)
     2. [Vascular Endothelial Growth Factor and Angiogenesis 15](#_TOC_250062)
     3. [The Role of VEGF and VEGF Receptor 2 in Angiogenesis and Tumor Growth 15](#_TOC_250061)
     4. [Ramucirumab 16](#_TOC_250060)
        1. [Clinical Studies 17](#_TOC_250059)
        2. [Rationale for the Use of Ramucirumab in the Treatment of Metastatic Gastric Carcinoma 17](#_TOC_250058)
        3. [Molecular classifications in GC 18](#_TOC_250057)
  5. [OBJECTIVES 18](#_TOC_250056)
     1. [Primary Objective 18](#_TOC_250055)
     2. [Secondary Objectives 18](#_TOC_250054)
  6. [POPULATION SELECTION 19](#_TOC_250053)
     1. [Inclusion Criteria 19](#_TOC_250052)
     2. [Exclusion Criteria 21](#_TOC_250051)
        1. [Rationale for Exclusion of Certain Patients 22](#_TOC_250050)
     3. [DISCONTINUATIONS 22](#_TOC_250049)
        1. [Discontinuation of Patients 22](#_TOC_250048)
  7. [CARE PLAN 23](#_TOC_250047)
     1. [Study Design and Plan 23](#_TOC_250046)
        1. [Pretreatment Period 24](#_TOC_250045)
        2. [Treatment Period 24](#_TOC_250044)
        3. [End of Therapy 25](#_TOC_250043)
        4. [Follow-Up 25](#_TOC_250042)
  8. [TREATMENT 25](#_TOC_250041)
     1. [Treatments Administered 25](#_TOC_250040)
        1. [Ramucirumab 25](#_TOC_250039)
        2. [Paclitaxel 25](#_TOC_250038)
     2. [Materials and Supplies 26](#_TOC_250037)
     3. [METHOD OF ASSIGNMENT TO TREATMENT 27](#_TOC_250036)
     4. [SELECTION AND TIMING OF DOSES 27](#_TOC_250035)
        1. [Special Treatment Considerations 27](#_TOC_250034)
     5. [CONCOMITANT THERAPY 28](#_TOC_250033)
  9. [EFFICACY AND SAFETY EVALUATION 30](#_TOC_250032)
     1. [EFFICACY 30](#_TOC_250031)
        1. [Molecular sequencing study. 30](#_TOC_250030)
     2. [SAFETY EVALUATIONS 30](#_TOC_250029)
        1. [Adverse Events 31](#_TOC_250028)
        2. [Infusion-related Reactions 34](#_TOC_250027)
        3. [Hypertension 35](#_TOC_250026)
        4. [Thrombotic Events 36](#_TOC_250025)
        5. [Bleeding (Hemorrhagic) Events 37](#_TOC_250024)
        6. [Proteinuria 37](#_TOC_250023)
        7. [Gastrointestinal Perforation 37](#_TOC_250022)
        8. [Reversible Posterior Leukoencephalopathy Syndrome (RPLS) 38](#_TOC_250021)
        9. [Congestive Heart Failure (CHF) 39](#_TOC_250020)
        10. [Impaired Wound Healing 39](#_TOC_250019)
        11. [Liver Failure 39](#_TOC_250018)
     3. [Appropriateness of Measurements 39](#_TOC_250017)
  10. [DATA QUALITY ASSURANCE 39](#_TOC_250016)
  11. [. SAMPLE SIZE AND STATISTICAL METHODS 39](#_TOC_250015)
      1. [Clinical Tests 40](#_TOC_250014)
         1. [Laboratory Parameters 40](#_TOC_250013)
         2. [Other Tests and Evaluations 41](#_TOC_250012)
      2. [Criteria for Tumor Response Evaluation 41](#_TOC_250011)
      3. [Symptomatic Deterioration (Clinical Progression) 41](#_TOC_250010)
      4. [Determination of Overall Response 41](#_TOC_250009)
  12. [INFORMED CONSENT, ETHICAL REVIEW AND REGULATORY CONSIDERATIONS](#_TOC_250008)

................................................................................................................................................. 41

- - 1. [Informed Consent 41](#_TOC_250007)
    2. [Ethical Review 41](#_TOC_250006)
    3. [Regulatory Considerations 42](#_TOC_250005)
    4. [Information for Treating Physicians 42](#_TOC_250004)
  1. [REFERENCES 43](#_TOC_250003)
  2. [APPENDIX 1 48](#_TOC_250002)
     1. [Post-Enrollment Evaluations: Frequency of Post-Enrollment Evaluations 48](#_TOC_250001)
  3. [Appendix 2 50](#_TOC_250000)

# LIST OF ABBREVIATIONS AND DEFINITIONS OF TERMS

5-FU 5-fluorouracil

AE adverse event

AESI adverse event of special interest

ALT alanine transaminase

ANC absolute neutrophil count

ASCO American Society of Clinical Oncology

AST aspartate transaminase

ATE arterial thrombotic events

β-HCG serum beta-human chorionic gonadotrophin (pregnancy test) BP blood pressure

BSC best supportive care

CF cisplatin + 5-fluorouracil

CFR Code of Federal Regulations

CHF congestive heart failure

complaint A complaint is any written, electronic, or oral communication that alleges deficiencies related to the identity, quality, purity, durability, reliability, safety or effectiveness, or performance of a drug or drug delivery system.

CR complete response

CRF case report form

CRP clinical research physician

CT computed tomography

CTD Clinical Trial Directive

DCSI Development Core Safety Information

DVT deep vein thrombosis

ECF epirubicin + cisplatin + 5-fluorouracil

ECOG PS Eastern Cooperative Oncology Group performance status ECX epirubicin + cisplatin + capecitabine

enroll The act of assigning a patient to treatment. Patients who are enrolled in the trial are those who have been assigned to treatment.

enter The act of obtaining informed consent for participation in a clinical trial from

patients deemed eligible or potentially eligible to participate in the clinicaltrial. Patients entered into a trial are those who sign the Informed Consent Form

directly or through their legally acceptable representatives.

EOF epirubicin + oxaliplatin + 5-fluorouracil

EOX epirubicin + oxaliplatin + capecitabine

GCP Good Clinical Practice

G-CSF granulocyte colony stimulating factor

GEJ gastroesophageal junction

GI gastrointestinal

HCC hepatocellular cancer

HIV human immunodeficiency virus

HR hazard ratio

IB Investigator’s Brochure

ICF informed consent form

ICH International Conference on Harmonisation

IDMC independent data monitoring committee

IgG_1_ immunoglobulin G, subclass 1

INR international normalized ratio

IP investigational product

IRB Institutional Review Board

IRR infusion-related reaction

I.V. intravenous

LV left ventricular

MAb monoclonal antibody

MAP mitogen-activated protein

MRI magnetic resonance imaging

MUGA multiple gated acquisition scan

NCI-CTCAE National Cancer Institute Common Terminology Criteria for Adverse Events NSAID nonsteroidal anti-inflammatory drug

OS overall survival

PD progressive disease

PE pulmonary embolism

PFS progression-free survival

PR partial response

PTT partial thromboplastin time

RECIST Response Evaluation Criteria in Solid Tumors RPLS reversible posterior leukoencephalopathy syndrome

SAE serious adverse event

SAER serious adverse event report

SD stable disease

SUSAR suspected unexpected serious adverse reaction TEAE treatment-emergent adverse event

TPO third party organization

TTP time-to-progression

UA urinalysis

ULN upper limit of normal

US United States

VEGF vascular endothelial growth factor VEGFR vascular endothelial growth factor receptor

VEGFR 2 vascular endothelial growth factor receptor-2 VTE venous thrombotic events

WOCBP woman (women) of childbearing potential

# INTRODUCTION

## Gastric Cancer

### Background

It is expected that more than 23,000 cases of gastric cancer (gastric adenocarcinoma) will have been diagnosed in the United States (US) in 2012, with approximately 10,500 deaths attributable to this form of malignancy (1). The incidence of this condition among white males living in the US increased more than 3.5-fold in the 20-year period beginning in 1974 (2). Within the European Union, there were an estimated 83,000 new cases of gastric cancer in 2008, and globally, there was an estimated 988,000 cases of gastric cancer diagnosed (3).

Internationally, the disease occurs with higher frequency among males. Gastric cancer incidence worldwide is more than double in men than in women (rate ratio 2.2:1.0). There is an 11-fold variation in male incidence rates between the regions of the world, and an 8-fold variation in female rates. In 2008, the highest incidence rates for both sexes were in Eastern Asia (42 and 18 per 100,000 in males and females, respectively), and the lowest were in Northern and Southern Africa (4 and 2 per 100,000 in males and females, respectively). The countries with the highest incidence rates in 2008 were Republic of Korea for males (62 per 100,000) and Guatemala and Republic of Korea for females (26 and 25 per 100,000, respectively) (4). Overall, gastric cancer is a major cause of cancer-related death worldwide (4), estimated to be responsible for a tenth, or nearly 740,000, of all cancer deaths in 2008. Gastric cancer mortality rates closely follow the trend for incidence rates (the ratio of mortality to incidence was 0.75 in 2008), with a similar variation in rates across the regions of the world (4).

### First-Line Therapy

While surgical resection is the preferred approach to treatment, approximately two-thirds of patients present with disease that is advanced or metastatic at diagnosis (6). For such patients, the prognosis is limited; the median survival for patients with untreated metastatic gastric cancer is 3 to 5 months (5,7,8,9). At present, systemic chemotherapy is the mainstay of treatment for advanced and metastatic gastric cancer (2). Combination chemotherapy, particularly containing fluoropyrimidines and platinum-based agents, has been shown to confer survival benefit compared with single-agent chemotherapy and supportive care (2,8). However, there have been

relatively few Phase 3 trials in this setting, and the studies that have been performed have been characterized by nonstandardized patient selection, methodology, and data monitoring processes, making it difficult to compare the results from disparate trials. For this reason, there is no single, accepted, first-line regimen for the treatment of advanced/metastatic gastric cancer (5).

In the last several decades, the incidence and prevalence of cancers of the gastroesophageal junction (GEJ), and particularly adenocarcinomas of the GEJ, have markedly increased (10). There is controversy as to whether such cancers should properly be classified as gastric, esophageal, or as a third independent entity, for the purposes of staging and research (11). In practice, systemic therapy for GEJ cancer has been based on that used for gastric cancer, and several major

studies have included both tumor types for the purposes of treatment analysis (2,12,13,14). Results of a large, recently conducted study involving 1002 patients with metastatic gastroesophageal cancers demonstrated that prognosis (in the metastatic setting) did not differ between gastric and GEJ adenocarcinomas (15).

In a 2008 report, Cunningham et al. described the results of a large, multicenter, Phase 3 study evaluating multiple strategies for the treatment of advanced esophagogastric cancer (15). Participants were randomized to 1 of 4 regimens:

- - - - epirubicin + oxaliplatin + 5-fluorouracil (5-FU) (EOF);
      - epirubicin + cisplatin + capecitabine (ECX);
      - epirubicin + cisplatin + 5-FU (ECF);
      - epirubicin + oxaliplatin + capecitabine (EOX).

No significant differences were observed in terms of response rate or progression-free survival (PFS). Overall survival (9.9 months, 9.3 months, 9.9 months, and 11.2 months for ECF, EOF, ECX, and EOX, respectively) was longer in patients receiving EOX versus ECF. These results demonstrate an efficacy of capecitabine equivalent to that of 5-FU and an efficacy of oxaliplatin equivalent to that of cisplatin.

A study published in 2006 by Van Cutsem et al. demonstrated that adding docetaxel to a regimen of cisplatin + 5-FU (CF) increased overall survival (OS), with the 2-year survival rate increasing to 18% from 9% compared with CF alone. However, the addition of docetaxel was associated with an increase in toxicity (5). Specifically, the addition of docetaxel was associated with increased Grade 3 and 4 neutropenia (82% versus 57% with CF alone), complicated neutropenia (29% versus 12%), Grade 3 and 4 diarrhea (19% versus 8%), and Grade 3 and 4 lethargy (19% versus 14%). Due in part to this increased toxicity, incorporation of docetaxel into first-line gastric cancer regimens has been limited.

Overall, none of the available first-line therapeutic regimens has been clearly established as a preferred option, and median survival associated with any of the available regimens remains limited. Almost all patients develop refractory disease; however, second-line treatment options are limited.

### Second-Line Therapy

A number of Phase 2 studies have been conducted in refractory gastric cancer. Published results are available from studies involving approximately 30 to 60 patients, almost exclusively from European and Asian centers and involving cytotoxic chemotherapy agents. The majority of patients had disease that was refractory to platinum-based therapy, although the range of prior therapies is extensive and also includes fluoropyrimidines, anthracyclines, and taxanes. Investigational therapies have included docetaxel, docetaxel/oxaliplatin, docetaxel/cisplatin, irinotecan plus

continuous-infusion 5-FU, irinotecan plus mitomycin-C, and mitomycin-C plus continuous- infusion 5-FU (13,14,16,17,18,19,20).

While these studies have demonstrated activity of a number of cytotoxic agents, they are not as informative as the randomized trials that have been reported over recent years.

The first of these randomized trials was a small German trial that stopped accrual prematurely due to the very low accrual seen (21). Notwithstanding a sample size of only 40 patients, the trial revealed patients on the treatment arm consisting of irinotecan every 3 weeks had a better survival than those allocated to best supportive care (BSC) with a hazard ratio (HR) of 0.48 (p=0.012). This corresponded to a median survival of 4.0 months on the irinotecan arm versus

2.4 months on the BSC arm. Grade 3 diarrhea occurred in 26% of patients on irinotecan, and febrile neutropenia was recorded in 3 of the 19 patients on this arm. No treatment-related deaths were seen. Toxicity was not reported for the control arm.

A Korean trial randomized 202 patients to BSC (n=69) or to BSC plus single-agent docetaxel or irinotecan, with the choice of chemotherapy left to the patient’s physician (n=133) (22). A survival advantage was seen with the active treatment arm; HR 0.657 (p=0.007, 1-sided) corresponding to a change in median survival from 3.8 months on BSC to 5.3 months on the chemotherapy arm. It is worth noting that 27% of patients in this study had received 2 lines of prior chemotherapy. In contrast to the German trial, side effects were reported for both arms, and the major side effects seen in the chemotherapy arm and not seen in the placebo arm were neutropenia, thrombocytopenia, and stomatitis. Diarrhea of any grade was seen in 14% and 15% of patients treated with docetaxel and irinotecan, respectively, but was also noted in 18% of patients on BSC. When considering Grade 3 or 4 diarrhea, the rates were 3% and 8% of patients treated with docetaxel and irinotecan, respectively, and 5% of patients on BSC. Of the 133 patients receiving chemotherapy, 6 non-fatal episodes of febrile neutropenia were seen. It is worth noting the German study enrolled patients with poorer performance status.

A global placebo-controlled randomized trial comparing an mTOR inhibitor (23) to BSC did not show an improvement in survival. Slightly over 50% of patients in this large 656 person trial had received 2 prior chemotherapy regimens.

A Japanese trial (24) randomized patients to weekly paclitaxel or biweekly irinotecan. While there was no statistically significant difference in survival, the better safety profile seen with the weekly paclitaxel regimen was accompanied by a numerically superior survival.

The REGARD study (25) compared ramucirumab, a human monoclonal antibody (MAb) targeting VEGF Receptor 2 to BSC in a placebo controlled randomized trial in which 238 patients were randomized to ramucirumab and 117 to placebo. The results revealed a statistically significant HR of 0.776 in favor of ramucirumab (p=0.047) with an improvement in median survival from 3.8 months to 5.2 months with ramucirumab exhibiting a tolerable safety profile without the side effects associated with cytotoxic chemotherapy. Table 1 depicts the adverse

events (AEs) reported from this trial and demonstrates a risk profile that differs from that seen with cytotoxic agents.

### Table 1. Adverse Events According to Grade in REGARD

|  | **Ramucirumab N = 236** | | **Placebo N =115** | |
| --- | --- | --- | --- | --- |
|  | **Any Grade**  **n (%)** | **≥Grade 3**  **n (%)** | **Any Grade**  **n (%)** | **≥Grade 3**  **n (%)** |
| Fatigue^a^ | 84 (36%) | 15 (6%) | 46 (40%) | 11 (10%) |
| Abdominal pain^b^ | 68 (29%) | 14 (6%) | 32 (28%) | 3 (3%) |
| Decreased appetite | 57 (24%) | 8 (3%) | 26 (23%) | 4 (3%) |
| Vomiting | 47 (20%) | 6 (3%) | 29 (25%) | 5 (4%) |
| Constipation | 36 (15%) | 1 (<1%) | 26 (23%) | 3 (3%) |
| Anemia^c^ | 35 (15%) | 15 (6%) | 17 (15%) | 9 (8%) |
| Dysphagia | 25 (11%) | 5 (2%) | 12 (10%) | 5 (4%) |
| Dyspnea | 22 (9%) | 4 (2%) | 15 (13%) | 7 (6%) |

Adverse events of special interest

| Hypertension^d^ | 38 (16%) | 18 (8%) | 9 (8%) | 3 (3%) |
| --- | --- | --- | --- | --- |
| Bleeding or hemorrhage^e^ | 30 (13%) | 8 (3%) | 13 (11%) | 3 (3%) |
| Arterial thromboembolism^f^ | 4 (2%) | 3 (1%) | 0 | 0 |
| Venous thromboembolism^g^ | 9 (4%) | 3 (1%) | 8 (7%) | 5 (4%) |
| Proteinuria | 7 (3%) | 1 (<1%) | 3 (3%) | 0 |
| Gastrointestinal perforation | 2 (<1%) | 2 (<1%) | 1 (<1%) | 1 (<1%) |
| Fistula formation | 1 (<1%) | 1 (<1%) | 1 (<1%) | 1 (<1%) |
| Infusion-related reaction | 1 (<1%) | 0 | 2 (2%) | 0 |
| Cardiac failure | 1 (<1%) | 0 | 0 | 0 |

Abbreviations: N = number of treated patients; n = number of patients in each category. a Includes asthenia.

b Includes upper or lower abdominal pain and hepatic pain. c Includes decreased hematocrit and red blood-cell count. d

Includes increased blood pressure.

1. Includes epistaxis, gastric hemorrhage, gastrointestinal hemorrhage, gingival bleeding, hematemesis, hematoma, hematuria, hemoptysis, hemorrhage, hemorrhoidal hemorrhage, melena, nail-bed bleeding,petechia, rectal hemorrhage, and upper gastrointestinal hemorrhage.
2. Includes angina pectoris, cardiac arrest, cerebral ischemia, cerebrovascular accident, myocardial infarction, and myocardial ischemia.
3. Includes pulmonary embolism, deep vein thrombosis, thrombosis, and venous thrombosis in a limb. Source: Adapted from Fuchs et al. 2014

The United Kingdom COUGAR-2 trial (26) randomized patients to docetaxel (n=84) or BSC (n=84) and reported a favorable improvement in survival; HR 0.67 (p=0.01) associated with an improvement in median survival from 3.6 to 5.2 months. Toxicity was reported to be the reason 31% of patients did not complete the targeted 6 cycles of docetaxel therapy.

Taken together, the results suggest chemotherapy or ramucirumab are reasonable therapeutic choices for patients with gastric cancer progressing after first-line therapy.

In January 2014 the results of the RAINBOW trial were reported (27). This international double-blind, placebo-controlled trial compared ramucirumab plus paclitaxel to placebo plus paclitaxel in patients with advanced gastric or GEJ adenocarcinoma whose disease had progressed during or after prior platinum and fluoropyrimidine chemotherapy. Paclitaxel was

administered at a dose of 80 mg/m^2^ on Days 1, 8, and 15 every 28 days in both arms, and ramucirumab was administered at a dose of 8 mg/kg every 2 weeks. Ramucirumab in combination with paclitaxel reduced the risk of death in this population by 19% (HR = 0.807; 95% confidence interval [CI]: 0.678, 0.962; p=0.0169), representing a 31% (2.3 months) longer median survival in the ramucirumab plus paclitaxel arm (9.63 months vs. 7.36 months in the placebo plus paclitaxel arm). Progression-free survival favored the ramucirumab plus paclitaxel arm (HR= 0.635; p<0.0001), as did objective response rate (28% vs. 16%; p=0.0001). The most significant differences in side effects occurred for neutropenia where the Grade 3 or higher rate in the ramucirumab plus paclitaxel arm was 40.7% versus 18.8% in the paclitaxel plus placebo arm. Grade 3 or worse hypertension was also markedly higher with a rate of 14.7% versus 2.7%. Despite the differences in neutropenia, rates of febrile neutropenia were similar (3.1% vs. 2.4%). Adverse events of special interest (AESIs) were numerically more common in the ramucirumab plus paclitaxel arm but apart from hypertension (all grades and ≥Grade 3), bleeding events (all grades only) and proteinuria (all grades only), the magnitude of the differences was modest.

More detailed information can be seen in Table 2 and Table 3.

### Table 2. Treatment-Emergent Adverse Events Occurring in ≥20% of Patients and

**≥5% Higher Incidence in the Ramucirumab Plus Paclitaxel Arm in RAINBOW**

|  | **Ramucirumab + Paclitaxel N = 327** | | **Placebo + Paclitaxel N =329** | |
| --- | --- | --- | --- | --- |
|  | **Any Grade**  **(%)** | **Grade ≥3**  **(%)** | **Any Grade**  **(%)** | **Grade ≥3**  **(%)** |
| Fatigue^a^ | 56.9 | 11.9 | 43.8 | 5.5 |
| Neutropenia^a^ | 54.4 | 40.7 | 31.0 | 18.8 |
| Neuropathy^a^ | 45.9 | 8.3 | 36.2 | 4.6 |
| Decreased appetite | 40.1 | 3.1 | 31.9 | 4.0 |
| Abdominal pain^a^ | 36.1 | 6.1 | 29.8 | 3.3 |
| Leukopenia^a^ | 33.9 | 17.4 | 21.0 | 6.7 |
| Diarrhea | 32.4 | 3.7 | 23.1 | 1.5 |
| Epistaxis | 30.6 | 0 | 7.0 | 0 |
| Vomiting | 26.9 | 3.1 | 20.7 | 3.6 |
| Hypertension^a^ | 25.1 | 14.7 | 5.8 | 2.7 |
| Peripheral edema | 25.1 | 1.5 | 13.7 | 0.6 |

Abbreviations: MedDRA = Medical Dictionary for Regulatory Activities; N = number of treated patients; n = number of patients in each category.

a Consolidated AE terms are comprised of synonymous MedDRA^®^ preferred terms: fatigue includes asthenia; neutropenia includes neutrophil count decreased; neuropathy includes peripheral sensory neuropathy; paresthesia; neuropathy peripheral, polyneuropathy; hypoesthesia, neuralgia, dysesthesia; abdominal pain includes abdominal pain upper and abdominal pain lower; leukopenia includes white blood cell decreased; hypertension includes blood pressure increased, hypertensive cardiomyopathy, procedural hypertension,

systolic hypertension.

Source: Adapted from Wilke et al. 2014

### Table 3. Adverse Events of Special Interest in RAINBOW

| Category of Event^a^ | **Ramucirumab + Paclitaxel N = 327** | | **Placebo + Paclitaxel N =329** | |
| --- | --- | --- | --- | --- |
|  | **Any Grade**  **(%)** | **Grade ≥3**  **(%)** | **Any Grade**  **(%)** | **Grade ≥3**  **(%)** |
| Bleeding/Hemorrhage | 41.9 | 4.3 | 17.9 | 2.4 |
| Epistaxis | 30.6 | 0 | 7.0 | 0 |
| Hypertension | 25.1 | 14.7 | 5.8 | 2.7 |
| Proteinuria | 16.8 | 1.2 | 6.1 | 0 |
| GI hemorrhage | 10.1 | 3.7 | 6.1 | 1.5 |
| Renal failure | 6.7 | 1.8 | 4.3 | 0.9 |
| Infusion-related reaction | 5.8 | 0.6 | 3.6 | 0 |
| Venous thromboembolism | 4.0 | 2.4 | 5.5 | 3.3 |
| Cardiac failure | 2.4 | 0.6 | 1.2 | 0.6 |
| Arteriothromboembolism | 1.8 | 0.9 | 1.5 | 0.9 |
| GI perforation | 1.2 | 1.2 | 0.3 | 0 |

Abbreviations: AESI = adverse event of special interest; GI = gastrointestinal; MedDRA = Medical Dictionary for Regulatory Activities; N = number of treated patients; n = number of patients in each category.

a Each AESI category is comprised of consolidated synonymous MedDRA® preferred terms. Source: Adapted from Wilke et al. 2014

## Vascular Endothelial Growth Factor and Angiogenesis

Angiogenesis, the formation of new capillaries and blood vessels, is a tightly controlled, multistep process that is a component of normal physiology (including development of the embryonic vasculature, wound healing, ovulation, and menstruation). Pathologic angiogenesis contributes to tumor growth and metastasis, as well as other human diseases, such as diabetic retinopathy, rheumatoid arthritis, and psoriasis (28,29,30). A number of growth factors have been identified as positive regulators of angiogenesis, including members of the vascular endothelial growth factor (VEGF) family, basic fibroblast growth factor, transforming growth factor alpha, transforming growth factor beta, tumor necrosis factor, platelet-derived endothelial growth factor, hepatocyte growth factor, angiogenin, interleukin-8, and placental growth factor (31,32).

## The Role of VEGF and VEGF Receptor 2 in Angiogenesis and Tumor Growth

The importance of VEGF and VEGF Receptor 2 in angiogenesis and tumor growth has been demonstrated in several animal models. Vascular endothelial growth factor-2 expression is associated with activated endothelium and is strongly upregulated in tumor endothelium (33,34).

Inhibiting the function of the VEGF/VEGF Receptor 2 pathway via a number of approaches, including anti-VEGF antibodies, anti-VEGF Receptor 2 antibodies, anti-VEGF antisense ribonucleic acid expression, VEGF-based immunotoxins, soluble VEGF receptors, ribozymes to VEGF receptors, and small molecule VEGF Receptor 2 tyrosine kinase inhibitors, has been shown to prevent new blood vessel formation and tumor growth in a variety of animal models (35,36,37,38).

Vascular endothelial growth factor and VEGF Receptor 2 are overexpressed in the great majority of human cancers, including carcinomas of the gastrointestinal (GI) tract, pancreas, breast, cervix, bladder, ovary, uterus, endometrium, and kidney; Kaposi’s sarcoma, glioblastoma multiforme, and hemangioblastomas. In addition, messenger ribonucleic acid for both VEGF Receptor 1 and VEGF Receptor 2 is greatly upregulated in tumor-associated endothelial cells, but not in the vasculature of the surrounding normal tissue. A correlation between VEGF Receptor 2 expression and tumor microvessel density has been associated with poor prognosis, advanced disease, increased risk of metastasis and recurrence, and lower relapse-free survival in patients with a variety of cancers (30,38).

## Ramucirumab

Ramucirumab is a recombinant human MAb of the immunoglobulin G, subclass 1 (IgG_1_) that specifically binds to the extracellular domain of VEGF Receptor 2 with high affinity. This antibody blocks the binding of the VEGF ligand to VEGF Receptor 2, inhibits VEGF-stimulated activation of both VEGF Receptor 2 and p44/p42 mitogen-activated protein (MAP) kinases, and neutralizes VEGF-induced mitogenesis of human endothelial cells.

Ramucirumab has been shown to block the interaction of VEGF and VEGF Receptor 2 (with a concentration that inhibits binding by 50% of approximately 1 nM), and to inhibit VEGF- stimulated proliferation of endothelial cells and VEGF-induced migration of human leukemia cells (39,40).

Preclinical pharmacodynamic data demonstrate that ramucirumab binds specifically and with high affinity to the VEGF Receptor 2 and is capable of inhibiting certain in vitro biological processes. These include VEGF/VEGF Receptor 2 interaction, VEGF-stimulated VEGF Receptor 2 activation, proliferation of human endothelial cells, VEGF-induced migration of

human leukemia cells, and VEGF-induced phosphorylation of VEGF Receptor 2 in both human umbilical vein endothelial cells and porcine aortic endothelial cells engineered to overexpress VEGF Receptor 2 (40). These processes are likely involved in tumor angiogenesis. Potent angiogenic and antitumor effects are observed when DC101, a rat antibody to murine VEGF Receptor 2, is administered to mice bearing syngeneic tumors or human tumor xenografts. The results of these preclinical pharmacodynamic studies support the investigation of ramucirumab in the treatment of solid tumors.

### Clinical Studies

A number of clinical studies have been completed and reported. Background material related to the early studies involving ramucirumab, its pharmacology, and toxicology may be found in the Investigator’s Brochure (IB). Clinical studies highlighted in this section relate to more recent studies involving ramucirumab.

In addition to the REGARD and RAINBOW studies, there are 4 other randomized Phase 3 trials involving ramucirumab. One of these, the ROSE trial, a double-blind randomized trial that compared ramucirumab in combination with docetaxel to placebo and docetaxel in the treatment of metastatic breast cancer, was negative as reported in December 2013 (41).

Another trial, REACH is comparing ramucirumab to placebo in second-line therapy of hepatocellular cancer (HCC). This study has raised the potential for drug-related liver injury/liver failure events based on independent Data Monitoring Committee (IDMC) interim safety analyses. While this observation has not been commented on by the DMCs for the other non-HCC Phase 3 trials, all ramucirumab protocols continuing to accrue patients have been amended to exclude patients with significant liver disease. This protocol will adopt this approach.

### Rationale for the Use of Ramucirumab in the Treatment of Metastatic Gastric Carcinoma

Vascular endothelial growth factor is expressed in gastric cancer, and expression has been associated with more aggressive clinical disease. Vascular endothelial growth factor expression has been noted in 51% of gastric cancer specimens in one series (versus no expression in normal epithelium or superficial gastritis) (42). Vascular endothelial growth factor expression in resected gastric cancer is associated with tumor recurrence and shorter survival. Maeda et al. studied 95 gastric cancer patients following resection with curative intent, and noted a significantly shorter survival in 34 patients whose tumor endothelium expressed VEGF (as detected via immunohistochemistry) versus 61 patients without endothelial VEGF expression (p<0.05) (43). Yoshikawa and colleagues observed similar survival differences in resected gastric cancer patients based on levels of circulating (plasma) VEGF at time of resection (44). Circulating VEGF is significantly higher in gastric cancer patients versus those without neoplasia. Elevated circulating VEGF was also associated with shorter survival in a European cohort undergoing gastric cancer resection; there was no survival beyond 30 months in 24 patients with serum VEGF

>533 pg/mL versus a 30-month survival rate >35% for 34 patients with VEGF levels below this threshold (p<0.0001, log-rank test) (45). Recently, Jüttner and colleagues noted reduced survival following R0 resection in gastric cancer patients whose tumors expressed VEGF-C or VEGF-D, with the most robust association between expression and reduced survival for patients whose tumors expressed both VEGF-C and VEGF-D (46).

Investigational inhibition of VEGF Receptor 2 in gastric cancer xenografts (TMK-1 cell line) is

associated with reduced tumor growth. DC101 therapy in this model is associated with significant reductions in tumor vascularity (as measured by CD-31 expression) and increases in endothelial and tumor apoptosis (47).

The results of the REGARD and RAINBOW studies are consistent with the idea that tumor- related angiogenesis contributes to the pathophysiology of gastric cancer and demonstrate the ability of ramucirumab to represent an improvement in the care of patients with gastric cancer whose disease has progressed after prior chemotherapy.

### Molecular classifications in GC

The molecular classification of GC and the relevance of pre-clinical models are not well established, creating challenges in discovering novel molecularly targeted therapies. In order to address these issues, we conducted an integrated molecular data analysis of three hundred Asian Gastric tumors through the Asian Cancer Research Group (ACRG). We performed an integrated genomic analyses based on target sequencing, gene expression profiling, copy number variations, Lauren’s histological classification, Epstein Barr Virus (EBV) status, TP53 status in three hundred GC specimens. We first divided GC into four subgroups based on gene expression profiling and TP53 status: 1) epithelial MSS-TP53 inactive; 2) epithelial MSS-TP53 active; 3) MSI; and 4) mesenchymal. With an integrative analysis with target sequencing and copy number variations, epithelial MSS-TP53 inactive GCs are characterized by predominantly hypermutated intestinal tumors (including majority of mutations in KRAS) with MLH1 loss through promoter methylation and MSS-TP53 active GCs are characterized by intact TP53 pathway with high frequency of EBV infection or frequently mutated oncogenes (e.g. PIK3CA). MSI subtype with TP53 pathway inactive characterized by TP53 loss through deleterious mutations in TP53 or MDM2 amplification and further characterized by both focal amplifications in oncogenes such as HER2, EGFR, cMET, CCNE1 as well as large scale chromosomal gains and losses. The above subtypes exhibited differential prognosis with the mesenchymal subtype displaying the worst survival (2.2 years) and the MSI subtype the most favorable survival (5.6 years). The GC subtypes and their association with prognosis were independently validated in three GC cohorts. In this study, we plan to analyze responders vs non- responders according molecular subtypes for ramucirumab.

# OBJECTIVES

## Primary Objective

To evaluate the response rate of Ramucirumab in combination with paclitaxel per RECIST 1.1

## Secondary Objectives

To analyze the response rate according to molecular subtypes identified through the ACRG effort,

with an integrative genomic analysis to identify predictive markers (i.e. angiogenesis signatures) for treatment response

# POPULATION SELECTION

Male and female patients with histologically or cytologically confirmed metastatic or locally recurrent unresectable gastric or GEJ adenocarcinoma and disease progression on prior fluoropyrimidine and/or platinum combination chemotherapy will be treated at the treatment centers. A record of the most recent pretreatment evaluations will be reviewed to determine the eligibility of a patient for this study.

While the eligibility criteria for REGARD and RAINBOW were similar, there were differences. Patients eligible for REGARD had to have prior therapy with a fluoropyrimidine or platinum combination chemotherapy, whereas patients on RAINBOW had to have received a platinum **and** fluoropyrimidine combination treatment Physicians with a specialty in oncology who have participated in an externally sponsored clinical trial within the previous 24 months may participate in this study. Since the prior chemotherapy requirements were slightly different between the 2 studies, this protocol will allow enrollment of patients who received either a platinum or a fluoropyrimidine or a platinum-fluoropyrimidine combination as first-line therapy for metastatic disease. The other area of difference is that patients in REGARD were eligible if the prior chemotherapy was given in the adjuvant setting provided such treatment was completed no more than 6 weeks prior to enrollment in the study. Patients in RAINBOW were to have all their prior chemotherapy given in the advanced or metastatic setting and not in the adjuvant setting. This protocol will restrict entry to those patients whose prior chemotherapy was given in the metastatic setting. Finally in RAINBOW while triplet chemotherapy was permissible as first- line therapy the additional agent had to be an anthracycline. The other commonly agent used as part of a triplet regimen is a taxane, notably docetaxel. This protocol will not impose this restriction of docetaxel use, leaving the decision to use a taxane again a matter of clinical judgment.

## Inclusion Criteria

Each patient must meet the following criteria to be enrolled in this study:

- - 1. The patient has histologically or cytologically confirmed gastric carcinoma, including gastric adenocarcinoma or GEJ adenocarcinoma. (Patients with adenocarcinoma of the distal esophagus are eligible if the primary tumor involves the GEJ.)
    2. The patient has metastatic disease or locally recurrent, unresectable disease.
    3. The patient has measureable or evaluable disease as determined by standard computed tomography (CT) or magnetic resonance imaging (MRI) imaging. Examples of evaluable, nonmeasurable disease include gastric, peritoneal, or mesenteric thickening in areas of known disease, or peritoneal nodules that are too small to be considered

measurable by Response Evaluation Criteria in Solid Tumors (RECIST version 1.1) (48).

- - 1. The patient has experienced disease progression during treatment or within 4 months after the last dose of first-line therapy for metastatic disease.
       - Acceptable prior chemotherapy regimens for this protocol are combination chemotherapy regimens that include platinum and/or fluoropyrimidine components (acceptable prior platinum agents are cisplatin, carboplatin, or oxaliplatin; acceptable prior fluoropyrimidine agents are 5-FU, capecitabine, or S-1). Regimens including a third agent, such as an anthracycline or a taxane, are acceptable provided a fluoropyrimidine and/or a platinum were used.
       - Recurrence during or within 6 months of completion of adjuvant chemotherapy (capecitabine, 5-FU, or TS-1) will be considered as first-line chemotherapy.
    2. The patient’s disease is not amenable to potentially curative resection.
    3. The patient is ≥18 years of age.
    4. The patient has resolution to Grade ≤1 (or to Grade ≤2 in the case of neuropathy) by the National Cancer Institute Common Terminology Criteria for Adverse Events (NCI- CTCAE), Version 4.03, of all clinically significant toxic effects of prior chemotherapy, surgery, radiotherapy, or hormonal therapy (with the exception of alopecia).
    5. The patient has an Eastern Cooperative Oncology Group performance status (ECOG PS) score of 0 or 1.
    6. The patient has adequate hepatic function as defined by a total bilirubin ≤1.5 mg/dL (25.65 µmol/L), and aspartate transaminase (AST) and alanine transaminase (ALT) ≤ 3.0 times the upper limit of normal (ULN; or 5.0 times the ULN in the setting of liver metastases).
    7. The patient does not have:
       - cirrhosis at a level of Child-Pugh B (or worse) or
       - cirrhosis (any degree) and a history of hepatic encephalopathy or clinically meaningful ascites resulting from cirrhosis. Clinically meaningful ascites is defined as ascites from cirrhosis requiring diuretics or paracentesis.
    8. The patient has adequate renal function as defined by a serum creatinine ≤1.5 times the ULN, or creatinine clearance (measured via 24-hour urine collection) ≥40 mL/minute (that is, if serum creatinine is >1.5 times the ULN, a 24-hour urine collection to calculate creatinine clearance must be performed).
    9. The patient’s urinary protein is ≤1+ on dipstick or routine urinalysis (UA; if urine dipstick or routine analysis is ≥2+, a 24-hour urine collection for protein must demonstrate <1000 mg of protein in 24 hours to allow participation in this protocol).
    10. The patient has adequate hematologic function, as evidenced by an absolute neutrophil count (ANC) ≥1000/µL, hemoglobin ≥9 g/dL (5.58 mmol/L), and platelets ≥100,000/µL.
    11. The patient must have adequate coagulation function as defined by international normalized ratio (INR) ≤1.5 and a partial thromboplastin time (PTT) ≤5 seconds above the ULN (unless receiving anticoagulation therapy). Patients on full-dose anticoagulation must be on a stable dose (minimum duration 14 days) of oral anticoagulant or low molecular weight heparin. If receiving warfarin, the patient must have an INR ≤3.0 and

no active bleeding (that is, no bleeding within 14 days prior to first dose of protocol therapy) or pathological condition present that carries a high risk of bleeding (for example, tumor involving major vessels or known varices). Patients on anticoagulation therapy with unresected primary tumors or local tumor recurrence following resection are not eligible.

- - 1. If the patient has received prior anthracycline therapy as part of his or her first-line regimen, the patient is able to engage in ordinary physical activity without significant fatigue or dyspnea (equivalent to New York Heart Association Class I function) (49).
    2. Because the teratogenicity of ramucirumab is not known, the patient, if sexually active, must be postmenopausal, surgically sterile, or using effective contraception (hormonal or barrier methods).
    3. Female patients of childbearing potential must have a negative serum pregnancy test within 7 days prior to enrollment.
    4. The patient is able to provide informed written consent.
    5. Feasible biopsy site

## Exclusion Criteria

Patients who meet any of the following criteria will be excluded from participation in this study:

- - 1. The patient has documented and/or symptomatic brain or leptomeningeal metastases.
    2. The patient has experienced any Grade 3 to 4 GI bleeding within 3 months prior to enrollment.
    3. The patient has experienced any arterial thromboembolic events, including but not limited to myocardial infarction, transient ischemic attack, cerebrovascular accident, or unstable angina, within 6 months prior to enrollment.
    4. The patient has an ongoing or active infection, symptomatic congestive heart failure, unstable angina pectoris, symptomatic or poorly controlled cardiac arrhythmia, uncontrolled thrombotic or hemorrhagic disorder, or any other serious uncontrolled medical disorders in the opinion of the treating physician.
    5. The patient has ongoing or active psychiatric illness or social situation that would limit compliance with treatment.
    6. The patient has uncontrolled or poorly controlled hypertension (>160 mmHg systolic or

>100 mmHg diastolic for >4 weeks) despite standard medical management.

- - 1. The patient has a serious or nonhealing wound, ulcer, or bone fracture within 28 days prior to enrollment.
    2. The patient has received chemotherapy, radiotherapy, immunotherapy, or targeted therapy for gastric cancer within 2 weeks prior to enrollment.
    3. The patient has received any investigational therapy within 30 days prior to enrollment.
    4. The patient has undergone major surgery within 28 days prior to enrollment, or subcutaneous venous access device placement within 7 days prior to enrollment.
    5. The patient has received prior therapy with an agent that directly inhibits VEGF (including bevacizumab), or VEGF Receptor 2 activity, or any antiangiogenic agent.
    6. The patient is receiving chronic antiplatelet therapy, including aspirin, nonsteroidal anti- inflammatory drugs (NSAIDs; including ibuprofen, naproxen, and others), dipyridamole or clopidogrel, or similar agents. Once-daily aspirin use (maximum dose 325 mg/day) is permitted.
    7. The patient has elective or planned major surgery to be performed during the course of the clinical trial.
    8. The patient has a known allergy to any of the treatment components.
    9. The patient is pregnant or breastfeeding.
    10. The patient is known to be positive for infection with the human immunodeficiency virus (HIV).
    11. The patient has known alcohol or drug dependency.
    12. The patient has a concurrent active malignancy other than adequately treated nonmelanomatous skin cancer, other noninvasive carcinoma, or in situ neoplasm.
    13. The patient has a known hypersensitivity to ramucirumab or any of the excipients.
    14. The patient may not have received more than 1 prior therapy in the metastatic setting.

### 6.2.1. Rationale for Exclusion of Certain Patients

All patients meeting the eligibility requirements will be eligible for enrollment regardless of race, religion, or sex. Patients who do not meet the enrollment criteria for the REGARD or RAINBOW studies will be excluded from this study as there is no information to support a favorable risk- benefit ratio for ramucirumab therapy for patients outside this target group.

Patients who meet all eligibility criteria will be made aware of the study, its specific aims and objectives, and the potential risks and benefits to patients associated with ramucirumab therapy in this disease state. See Section 13.1 for details on informed consent. There will be no financial compensation for patients enrolling in this study. Pretreatment evaluations (see Post- Enrollment Evaluations [Appendix 1]) must be sufficient to ensure patients meet all eligibility requirements and sufficient to adequately assess disease control during the course of care. The treating physician will telephone the call center to enroll patients in the study.

## DISCONTINUATIONS

### Discontinuation of Patients

The criteria for enrollment must be followed explicitly. If the site identifies a patient who did not meet enrollment criteria and who was inadvertently enrolled, the sponsor or designee must be notified. If the sponsor or designee identifies a patient who did not meet enrollment criteria and who was inadvertently enrolled, the site will be notified.

In addition, patients will be discontinued from the study in the following circumstances:

- - - - enrollment in any clinical trial involving an IP or enrollment in any other type of medical research judged not to be scientifically or medically compatible with this study
      - treating physician decision
        - the treating physician decides that the patient should be discontinued from the

study

- - - - - if the patient, for any reason, requires treatment with another therapeutic agent that has been demonstrated to be effective for treatment of the patient’s gastric or GEJ cancer, discontinuation from the ramucirumab occurs prior to introduction of the new agent
      - patient decision
        - the patient (or the patient’s legally acceptable representative) requests to be withdrawn from the study

# CARE PLAN

## Study Design and Plan

This study is a single arm, open label, single center phase II study of ramucirumab in combination with paclitaxel in patients with advanced gastric adenocarcinoma including gastric adenocarcinoma or GEJ adenocarcinoma as a second line chemotherapy

Patients will receive ramucirumab plus weekly paclitaxel combination regimen. This study recruits a maximum of 61 patients.

Lilly will only supply ramucirumab and no other medications used in the care of patients while on this study.

Patients will receive ramucirumab, administered via intravenous (I.V.) infusion over approximately 1 hour, every 2 weeks, at a dose of 8 mg/kg,

Paclitaxel will be administered at a dose of 80 mg/m^2^ I.V. infusion on Days 1, 8, 15 of a 28-day cycle.

Assessment of disease control is recommended every 6 weeks.

It is recognized that in the course of clinical cancer care, it is not always possible to schedule therapeutic infusions precisely 2 weeks following a prior infusion (because of holidays, travel difficulties, or other circumstances). Accordingly, infusions administered within 3 days before or after the planned 2-week time point are acceptable. Administration beyond this window is strongly discouraged.

All patients may continue to receive treatment with the IP, ramucirumab, until there is evidence of progressive disease (PD), unacceptable toxicity, the patient is withdrawn from the study for any other reason, or ramucirumab becomes commercially available and accessible to patients after local marketing authorization.

Treatment may be discontinued as outlined in Section 7.3.3.

### Pretreatment Period

Evaluations to determine patient eligibility for this study should be conducted within 28 days of enrollment or closer to the time of treatment if the patient’s clinical condition has changed. Written informed consent must be obtained before patient enrollment (see Section 13.1). Standard of care laboratory samples (blood or urine) collected prior to the signing of the consent form may be used as screening samples provided they were collected within 28 days of the first dose of protocol therapy.

All patients must undergo appropriate evaluations in order to ensure the patients meet all eligibility criteria.

Results of all pretreatment evaluations must be reviewed by the attending physician or his/her designee to ensure that all eligibility criteria have been satisfied prior to patient enrollment.

Screening numbers and Enrollement numbers will be assigned as follows.

-Screening number: S001,S002,S003…

-Enrollment number: E001, E002, E003….

### Treatment Period

Patients will be assessed by physical examination and ancillary tests according to local practice. Prior to each dose of ramucirumab, it is recommended that patients are assessed clinically, including blood pressure (BP) assessment and laboratory tests, to ensure it is appropriate to continue therapy. Vital sign measurements, including temperature, pulse rate, respiration rate, and BP, should be obtained before, during, and at the completion of each infusion of ramucirumab.

The Post-Enrollment Evaluations (Appendix 1) summarizes all assessments, tests, and treatments that should occur as part of this study. In situations where hemoglobin is less than 9 g/dL (5.58 mmol/L) and there are signs or symptoms of bleeding, a hematology profile should be performed weekly until hemoglobin is ≥9 g/dL (5.58 mmol/L) and any bleeding-related signs or symptoms have resolved or been adequately investigated.

As noted in the Post-Enrollment Evaluations, additional evaluations should be performed every 6 weeks prior to submitting a reorder for ramucirumab and should include:

- serum beta-human chorionic gonadotrophin (β-HCG) pregnancy test in women of

childbearing potential (WOCBP; every 6 weeks or in accordance with local regulations, whichever is of shorter duration);

- imaging or other evaluations as appropriate to assess disease control;
- hematology, chemistry, and coagulation profiles;
- UA;
- BP assessment.

### End of Therapy

The following evaluations are recommended when patients discontinue treatment with ramucirumab:

- - - - hematology, chemistry, and coagulation profiles;
      - imaging studies with tumor measurements/disease response assessments if the patient was discontinued for reasons other than PD;
      - pregnancy test for WOCBP;
      - UA;
      - BP assessment.

### Follow-Up

Follow-up and treatment of patients postdiscontinuation of ramucirumab is at the discretion of the treating physician. The attending physician will be asked to complete the final case report form (CRF) indicating whether he/she believed the patient derived benefit from ramucirumab and whether there are plans to commence another form of systemic therapy. The completion of this form will officially indicate no further ramucirumab will be requested for this particular patient.

# TREATMENT

## Treatments Administered

### Ramucirumab

Different drug product lots must not be mixed in a single infusion.

Patients will receive 8 mg/kg of ramucirumab every 2 weeks, administered as an I.V. infusion over approximately 1 hour (maximum infusion rate 25 mg/min). The dose of ramucirumab will be dependent upon the patient’s baseline body weight in kilograms. This dose will be recalculated if there is a ≥10% change in body weight from baseline. Refer to the IB for more details.

### Paclitaxel

Treating physicians should consult the manufacturer’s instructions for paclitaxel for complete prescribing information (including warnings, precautions, contraindications, and adverse reactions) and follow institutional procedures for the administration of paclitaxel.

Aseptic technique is to be used when preparing and handling paclitaxel. Patients will receive paclitaxel by I.V. infusion over approximately 60 minutes at 80 mg/m^2^ on Days 1, 8, and 15 of every 28-day cycle.

Patients receiving paclitaxel may experience hypersensitivity reactions. The treating physician should institute appropriate treatment measures according to local medical practice. The following treatment guidelines may be applicable:

For mild symptoms: Complete paclitaxel infusion. Supervise at bedside. No treatment required.

For moderate symptoms: Stop paclitaxel infusion. Administer diphenhydramine 25 to 50 mg

I.V. and dexamethasone 8-20 mg I.V. (or equivalent, per institutional guidelines). Resume the paclitaxel infusion after recovery of symptoms at a reduced rate (20 mL/hour for 15 minutes). The infusion rate may then be increased to 40 mL/hour for 15 minutes, and subsequently at the full rate if symptoms do not recur. If symptoms recur, stop the paclitaxel infusion and remove patient from paclitaxel treatment.

For severe life-threatening symptoms: Stop paclitaxel infusion. Give I.V. diphenhydramine and dexamethasone (per institutional guidelines). Add epinephrine or bronchodilators if indicated. The patient should be removed from paclitaxel treatment.

## Materials and Supplies

For instructions on reconstitution and administration of ramucirumab, refer to the IB (Safe Handling and Administration). Calculate the dose and volume of ramucirumab needed to prepare the infusion solution. Vials contain either 100 mg or 500 mg as a 10 mg/mL solution of ramucirumab. Only use sterile sodium chloride (0.9%) solution for injection as a diluent.

Administer via an infusion pump. A separate infusion line must be used for the infusion and the line must be flushed with sterile sodium chloride (0.9%) solution for injection at the end of the infusion.

As infusion-related reactions (IRRs) may occur during or after the administration of ramucirumab, premedication is recommended with a histamine H1 antagonist (such as, diphenhydramine hydrochloride or equivalent) intravenously prior to administration of ramucirumab.

If a patient experiences a Grade 1 or 2 IRR, premedication must be given for all subsequent infusions. If a patient has a second Grade 1 or 2 IRR, administer dexamethasone (or equivalent);

then, for subsequent infusions, premedicate with diphenhydramine hydrochloride (or equivalent) (see Section 10.2.1.3).

Vials should be stored in a refrigerator at 2°C to 8°C (36°F to 46°F) until time of use. Keep the vial in the outer carton in order to protect from light. DO NOT FREEZE OR SHAKE the vial.

The chemical and physical stability for the ramucirumab infusion solution was demonstrated for up to 24 hours at 2°C to 8°C (36°F to 46°F) or for 4 hours at room temperature (below 25°C (77°F)). DO NOT FREEZE OR SHAKE the ramucirumab infusion solution.

## METHOD OF ASSIGNMENT TO TREATMENT

This is a single-arm study and all patients enrolled will receive ramucirumab/paclitaxel.

## SELECTION AND TIMING OF DOSES

Patients will be treated according to the regimen used in the REGARD and RAINBOW studies in which ramucirumab was administered at a dose of 8 mg/kg every 2 weeks, administered as an

I.V. infusion over approximately 1 hour (maximum infusion rate 25 mg/min). The dose will be dependent upon the patient’s baseline body weight in kilograms and will be recalculated if there is a ≥10% change in body weight from baseline.

### Special Treatment Considerations

- - - 1. Dose Adjustments and Delays

Dose modifications are permitted for ramucirumab in the setting of non-life-threatening, reversible Grade 3 and 4 AEs (that is, fatigue, anorexia, or fever) that resolve to Grade ≤1 within 1 treatment cycle (approximately 2 weeks). In this setting, ramucirumab may be readministered. If a second instance of such an event occurs, ramucirumab should be subsequently readministered at a dose of 6 mg/kg every other week. A second dose reduction to 5 mg/kg every other week is permitted for this level of event (Grade 3 and 4). If a Grade 4 AE occurs and is deemed at least possibly related to ramucirumab, then ramucirumab should be discontinued except in specific case of Grade 4 fever or Grade 4 laboratory abnormalities. If Grade 4 fever or laboratory abnormalities resolve to Grade ≤1 or pretreatment baseline within one treatment cycle (approximately 2 weeks), treatment with ramucirumab may be continued at the discretion of the treating physician. Criteria for dose reduction in the setting of hypertension or proteinuria and other AESIs are detailed in Section 10.2.1.3. Criteria for discontinuation of ramucirumab therapy are detailed in Section 7.3.1.

Patients who enter the study with symptoms or laboratory values equivalent to NCI-CTCAE Version 4.03 Grade 1 to 2 AEs should not have dose reductions related to the persistence or mild worsening of those symptoms or laboratory values; dose reductions may be warranted if

worsening of symptoms or laboratory values is clinically significant in the opinion of the patient’s physician. Asymptomatic Grade 3 to 4 laboratory abnormalities should not result in dose interruptions, modifications, or discontinuation of protocol therapy unless determined to be clinically significant by the patient’s physician (or unless otherwise specified in this protocol).

The treatment schedule remains static regardless of interruptions in ramucirumab administration. Dose delays are only permitted within 3 days after the date of a regularly scheduled treatment. If a patient is unable to receive treatment on or within 3 days before or after the date of his/her regularly scheduled dose for any reason (including AEs), the next dose of ramucirumab should be administered at the first regularly scheduled treatment time point following the resolution of the event causing the delay. (Treatment in this situation [missed dose/cycle] is considered to occur at the subsequently numbered cycle.) Make-up doses occurring between regularly scheduled, every-2-week treatment time points are not recommended.

The patient’s physician may discontinue ramucirumab for lack of disease control or toxicity. Patients who experience an AE requiring dose reduction after 2 prior dose reductions should discontinue ramucirumab therapy. In addition, the patient’s physician may withdraw a patient from ramucirumab for disease progression, toxicity, or if the treating physician team determines it is in the patient’s best interests for any reason. Patients whose performance status worsens by

>2 units should be discontinued from study therapy.

After termination of ramucirumab therapy, the patient will be treated as clinically indicated by the treating physician.

- - - 1. Hematologic Toxicity

Reduced leukocyte or platelet counts are not anticipated ramucirumab-related AEs. However, on the planned day of treatment, ramucirumab should only be administered if:

- - - - - ANC is ≥1000/µL; and
        - platelet count is ≥75,000/µL.

## CONCOMITANT THERAPY

Palliative and supportive care for other disease-related symptoms and for toxicity associated with treatment will be offered to all patients on this study. This may include but is not limited to antiemetic agents, opiate and nonopiate analgesic agents, appetite stimulants, and granulocyte and erythroid growth factors. Medical teams are advised to prescribe these agents according to American Society of Clinical Oncology (ASCO) or local guidelines.

The use of other chemotherapy, experimental medications, other anticancer therapy, immunotherapy, hormonal cancer therapy, radiation, surgery for cancer, or experimental medications is indicative that the patient’s disease or clinical condition is worsening and should

be discontinued from this study. Combining any other form of anticancer therapy with ramucirumab is strongly discouraged as there is no information supporting that this can be done safely in this clinical situation.

Ramucirumab has not been studied in conjunction with radiation. Therefore, it is recommended that sites contact the lead Lilly CRP or designate prior to administering palliative radiation. If palliative radiation is being administered to new symptomatic areas, this may represent progression of disease in which case the patient should discontinue ramucirumab therapy. Patients should not receive chronic antiplatelet therapy, including NSAIDs (including ibuprofen, naproxen, and others), dipyridamole or clopidogrel, or similar agents.

Aspirin is permitted at doses ≤325 mg once daily. Ongoing aspirin therapy at doses exceeding 325 mg/day is not permitted.

Anticoagulation therapy is permitted as follows:

At entry into the protocol, patients on full-dose anticoagulation must be on a stable dose (minimum duration 14 days) of oral anticoagulant or low molecular weight heparin. If on warfarin, the patient must have an INR ≤3 and no active bleeding or pathological condition present that carries a high risk of bleeding (for example, tumor involving major vessels or known varices).

Patients who develop venous thromboembolism during protocol therapy may continue study therapy but must receive low molecular weight heparin, not oral anticoagulation.

The effects of ramucirumab on wound healing are not known. Bevacizumab (an antibody to VEGF-A ligand) is associated with a higher incidence of serious wound-healing complications.

In one study, 6 of 39 patients (15%) requiring surgery during or following bevacizumab experienced complications related to wound healing or bleeding; in a second study, complications were experienced by 1 of 25 patients (4%) requiring surgery (50). Hence, it is recommended that major surgery either be performed more than 28 days prior to enrollment or be postponed until at least 28 days after last dose of ramucirumab, when possible, and that subcutaneous venous access devices be placed at least 7 days prior to enrollment if their use is likely to be warranted. If major surgery is required during study therapy, the surgeon should be informed that the patient is receiving an agent that may be associated with a higher incidence of wound-healing complications. If subcutaneous venous access device placement is required during the course of protocol therapy, it is recommended that a 7-day treatment-free period occur both prior to and following placement.

Although emesis is not an expected adverse drug reaction to ramucirumab, the use of antiemetic agents is permitted at the discretion of the treating physician.

The use of analgesic agents is permitted at the discretion of the treating physician. Opiate and

nonopiate analgesic agents are permitted (including acetaminophen); however, the use of NSAIDs and/or aspirin is prohibited as detailed above.

The use of appetite stimulants is permitted at the discretion of the treating physician.

Although neutropenia is not an expected adverse drug reaction of ramucirumab, the use of granulocyte colony stimulating factors (G-CSF) is permitted in accordance with ASCO or local guidelines.

The use of erythroid-stimulating factors (for example, erythropoietin) is permitted in accordance with ASCO or local guidelines.

The use of benzodiazepines, antidepressants, laxatives, and other agents that may be helpful in controlling disease-related symptoms are also permitted and encouraged, except as prohibited as outlined above.

# EFFICACY AND SAFETY EVALUATION

Written informed consent must be obtained prior to any study -specific pretreatment evaluations (see Section 13.1). Study procedures related to assessments and their timing are described in the sections below and shown in the Post-Enrollment Evaluations (Appendix 1).

## EFFICACY

Patients enrolled in this study must have either measureable or evaluable disease in order to objectively evaluate the benefits of ramucirumab therapy. Patients should have repeat imaging of their disease every 6 weeks or at a time interval consistent with local institutional practice.

### Molecular sequencing study.

All patients enrolled onto this trial will be subject to sequencing project. Baseline and follow up biopsy at progression will be collected. DNA/RNA sequencing will be performed at designated sequencing vendor. Sequencing will be targeted/whole exome sequencing + RNA sequencing. The data will be shared by Lilly and SMC. Publication will be a joint publication with major authors being from SMC, but possibly from Lilly depending on scientific contribution. A separate genomic screening test according to the Korean law will be obtained in all patients and tissue specimens will be handled accordingly.

## SAFETY EVALUATIONS

Treating physicians are responsible for monitoring the safety of patients who have entered this study and for alerting Lilly or its designee to any event that seems unusual, even if this event may be considered an unanticipated benefit to the patient.

The treating physician is responsible for the appropriate medical care of patients while on this study.

The treating physician remains responsible for following, through an appropriate health care option, AEs that are serious, considered related to care received while on this study, or that caused the patient to discontinue from the study. The patient should be followed until the event is resolved or explained. The frequency of follow-up evaluation is left to the discretion of the patient’s physician.

### Adverse Events

**SMC will follow Lilly’s SAE reporting system in investigator sponsored trials.**

Lack of drug effect is not an AE in this study.

Cases of pregnancy that occur during maternal or paternal exposures to ramucirumab should be reported. Data on fetal outcome and breastfeeding are collected for regulatory reporting and drug safety evaluation.

Site personnel will record in the patient’s medical record the occurrence and nature of each patient’s preexisting conditions, including clinically significant signs and symptoms of the disease under treatment.

After the ICF is signed, site personnel will record the occurrence and nature of any AEs targeted for collection in this study. Information on the AEs collected during the patient’s time on the Study will be reported to Lilly or its designee at the time the patient discontinues ramucirumab. Adverse events must also be documented at the time of discontinuation and for 30 days after last dose of protocol therapy.

The treating physician will decide whether he or she interprets the recorded AEs as related to disease, study medication, study procedure, or other concomitant treatment or pathologies. To assess the relationship of the AE to the study drug or procedure, the following terminologies are defined:

- - - - **Probably related**: A direct cause and effect relationship between the study treatment and the AE is likely.
      - **Possibly related**: A cause and effect relationship between the study treatment and the AE has not been demonstrated at this time and is not probable, but is also not impossible.
      - **Does not know**: The treating physician cannot determine.
      - **Not related**: Without question, the AE is definitely not associated with the study treatment.

The treating physician should classify all “probably related,” “possibly related,” or “does not know” AEs and SAEs as related to study drug/study procedure.

Patients will be evaluated for AEs at each visit and will be instructed to call their physician to report any AEs between visits.

The NCI-CTCAE (v4.03) will serve as the reference document for choosing appropriate terminology for, and grading the severity of, all AEs and other symptoms. For AEs without matching terminology within the NCI-CTCAE (v4.03) criteria, the treating physician will be responsible for selecting the appropriate system organ class and assessing severity grade based on the intensity of the event.

Apart from SAEs (Section 10.2.1.1) and AESIs (Section 10.2.1.3), only those AEs Grade 3 or worse will be reported at the time the patient discontinues ramucirumab therapy.

- - - 1. Serious Adverse Events

A serious adverse event is any AE from this study that results in one of the following outcomes:

- - - - - death
        - a life-threatening experience (that is, immediate risk of dying)
        - persistent or significant disability/incapacity
        - initial or prolonged inpatient hospitalization
        - congenital anomaly/birth defect
        - considered significant by the investigator for any other reason

Important medical events that may not result in death, be life-threatening, or require hospitalization may be considered SAEs when, based upon appropriate medical judgment, they may jeopardize the patient and may require medical or surgical intervention to prevent one of the outcomes listed in this definition.

Serious adverse event collection begins after the patient has signed informed consent and has received ramucirumab. If a patient experiences an SAE after signing informed consent, but prior to receiving ramucirumab, the event will not be reported as an SAE unless the investigator feels the event may have been caused by a protocol required procedure. Serious adverse events must also be documented at the time of discontinuation and for 30 days after last dose of protocol therapy. Serious adverse events that occur more than 30 days after last dose of protocol therapy must be reported if deemed related to protocol procedures or study drug by the patient’s physician.

Study personnel must alert Lilly or its designee of any SAE within 24 hours of treating physician awareness of the event via a sponsor-approved method. If alerts are issued via telephone, they are to be immediately followed with official notification on study-specific SAE forms.

This 24-hour notification requirement refers to the initial SAE information and all follow-up SAE information.

Planned surgeries should not be reported as SAEs unless the underlying medical condition has worsened after commencing ramucirumab therapy.

Planned hospitalizations or procedures for preexisting conditions that are already recorded in the patient’s medical history at the time of study enrollment should not be considered SAEs. Hospitalization or prolongation of hospitalization without a precipitating clinical AE (for example, for the administration of study therapy or other protocol-required procedure) should not be considered SAEs.

Death due to disease progression should not be reported as an SAE unless the treating physician also deems there to be a contribution possibly related to the study drug.

If a treating physician becomes aware of an SAE occurring after the patient’s participation in the study has ended, and the treating physician believes that the SAE is related to ramucirumab, the treating physician should report the SAE to the sponsor, and the SAE will be entered in the sponsor’s pharmacovigilance system.

Information on SAEs expected in the study population independent of drug exposure and that will be assessed by the sponsor in aggregate periodically during the course of the study may be found in the IB.

**All** SAEs identified while a patient is on this study will be reported to Lilly as noted above.

- - - 1. Suspected Unexpected Serious Adverse Events

Suspected unexpected serious adverse reactions (SUSARs) are serious events that are not listed in the Development Core Safety Information (DCSI) in the IB and that the treating physician identifies as related to the IP or study procedure. United States 21 Code of Federal Regulations (CFR) 312.32 and European Union Clinical Trial Directive (CTD) 2001/20/EC and the associated detailed guidances or national regulatory requirements in participating countries require the reporting of SUSARs. Lilly has procedures that will be followed for the recording and expedited reporting of SUSARs that are consistent with global regulations and associated detailed guidances.

- - - 1. Adverse Events of Special Interest (AESIs)

Adverse events of special interest, which may or may not be associated with ramucirumab therapy, include infusion-related reactions, hypertension, arterial or venous thrombotic events, bleeding (hemorrhagic) events, proteinuria, gastrointestinal perforation, reversible posterior leukoencephalopathy syndrome (RPLS), congestive heart failure (CHF), impaired wound healing, and liver failure and other significant liver injury. The worst grade of each of these events, if they occurred, will be reported on the designated CRF at the time the patient discontinues

ramucirumab therapy.

### Infusion-related Reactions

Infusion-related reactions are defined according to the NCI-CTCAE Version 4.03 definition of allergic reaction/hypersensitivity, as follows:

- - - - Grade 1: Mild, transient reaction; infusion interruption not indicated; intervention not indicated
      - Grade 2: Therapy or infusion interruption indicated but responds promptly to symptomatic treatment (for example, antihistamines, NSAIDS, narcotics, I.V. fluids); prophylactic medications indicated for ≤24 hours
      - Grade 3: Prolonged (for example, not rapidly responsive to symptomatic medication and/or brief interruption of infusion); recurrence of symptoms following initial improvement; hospitalization indicated for clinical sequelae
      - Grade 4: Life-threatening consequences; urgent intervention indicated

Consistent with usual medical practice, selected parenteral medications may be utilized for Grade 2 allergic/hypersensitivity reaction as detailed below.

The following are suggested treatment guidelines for all infusion-related reactions: Grade 1 Slow the infusion rate by 50%.

- - - - Monitor the patient for worsening of condition.
      - For subsequent infusions, premedicate with diphenhydramine hydrochloride 50 mg I.V. (or equivalent); additional premedication may be administered at the discretion of the patient’s physician.

Grade 2

- - - - Stop the infusion.
      - Administer diphenhydramine hydrochloride 50 mg I.V. (or equivalent), acetaminophen 650 mg orally for fever, and oxygen.
      - Resume the infusion at 50% of the prior rate once the infusion reaction has resolved or decreased to Grade 1; the infusion duration should not exceed 2 hours.
      - Monitor for worsening of condition.
      - For subsequent infusions, premedicate with diphenhydramine hydrochloride 50 mg I.V. (or equivalent); additional premedication may be administered at the discretion of the patient’s physician.

For a second Grade 1 or 2 infusion reaction, administer dexamethasone 8-10 mg I.V. (or equivalent); then, for subsequent infusions, premedicate with diphenhydramine hydrochloride 50 mg I.V. (or equivalent), acetaminophen 650 mg orally, and dexamethasone 8-10 mg I.V. (or

equivalent). Grade 3

- - - - Stop the infusion and disconnect the infusion tubing from the patient.
      - Administer diphenhydramine hydrochloride 50 mg I.V. (or equivalent), dexamethasone 8-

10 mg I.V. (or equivalent), bronchodilators for bronchospasm, and other medications/treatment as medically indicated.

- - - - Patients who have a Grade 3 infusion reaction must not receive further treatment with ramucirumab.

Grade 4

- - - - Stop the infusion and disconnect the infusion tubing from the patient.
      - Administer diphenhydramine hydrochloride 50 mg I.V. (or equivalent), dexamethasone 8-10 mg I.V. (or equivalent), and other medications/treatment as medically indicated.
      - Give epinephrine or bronchodilators as indicated.
      - Hospital admission for observation may be indicated.
      - Patients who have a Grade 4 infusion reaction must not receive further treatment with ramucirumab and will be cared for at the discretion of the treating physician.

### Hypertension

If a patient develops hypertension while on protocol therapy, he or she should be treated with antihypertensive medications according to standard medical practice.

Grade <3

- - - - For controlled hypertension (<160/100 mm Hg), ramucirumab therapy should continue without interruption.
      - For asymptomatic hypertension, continue ramucirumab with initiation of antihypertensive therapy.
      - If the hypertension is associated with symptoms, hold ramucirumab until symptoms resolve and initiate antihypertensive therapy.

If ramucirumab is held for hypertension (ie, symptomatic hypertension, markedly elevated BP unresponsive to antihypertensive therapy), the dose of ramucirumab should be reduced to 6 mg/kg every other week upon re-treatment. A second dose reduction to 5 mg/kg every other week should be undertaken if an additional (third) postponement of therapy is required.

Grade 3 (systolic BP ≥160 mm Hg or diastolic BP ≥100 mm Hg; medical intervention indicated:

more than 1 drug or more intensive therapy than previously used indicated)

- - - - For Grade 3 hypertension not associated with symptoms, continue ramucirumab with more intensive antihypertensive therapy. If systolic BP remains >160 mm Hg or diastolic BP >100 mm Hg more than 2 weeks after initiation of additional antihypertensive therapy (as noted above), study ramucirumab will be held while continuing appropriate antihypertensive therapy.
      - If hypertension is associated with symptoms, hold ramucirumab until symptoms resolve and initiate antihypertensive therapy.

If ramucirumab is held more than once for hypertension (ie, symptomatic hypertension, markedly elevated BP unresponsive to antihypertensive therapy), the dose of ramucirumab should be reduced to 6 mg/kg every other week upon re-treatment. A second dose reduction to 5 mg/kg every other week should be undertaken if an additional (third) postponement of therapy is required.

Grade 4 or refractory

- Patients with Grade 4 hypertension (life-threatening consequences; eg malignant hypertension, transient or permanent neurologic deficit, hypertensive crisis; or urgent intervention indicated) or patients whose hypertension is poorly controlled (>160 mm Hg systolic or >100 mm Hg diastolic BP for >4 weeks) despite appropriate oral medication (>2 oral agents at maximum tolerated dose) will be discontinued from therapy.

### Thrombotic Events

Physicians should perform all testing required to fully characterize arterial or venous thrombotic/vascular events. In the REGARD study, venous thrombotic events (VTEs) were more frequent on the placebo arm than on ramucirumab (all grade 7.0% versus 3.8%; and Grade 3 or greater 4.3% and 1.3%, respectively). Arterial thrombotic events (ATEs) occurred in patients treated with ramucirumab (1.7% all grade; 1.3% Grade 3 or greater) and in no patients treated with placebo. In RAINBOW, the overall incidence of all grade and Grade ≥3 VTEs and arterial thromboembolic events were low and similar in both treatment arms (ramucirumab plus paclitaxel vs. placebo plus paclitaxel: all grade VTEs [4.0% vs. 5.5%] and Grade 3 or greater VTEs [2.4% vs. 3.3%]; all grade ATEs [1.8% vs. 1.5%] and Grade 3 or greater ATEs [0.9% vs.

0.9%]).

Because therapy with antiangiogenic agents has been shown to be feasible in the setting of anticoagulation in patients with upper GI malignancies (12), patients who develop Grade 3 and 4 venous thrombotic events (deep vein thrombosis [DVT] or pulmonary embolism [PE]) may continue ramucirumab therapy if the event is not considered to be life-threatening in the opinion of the patient’s physician, the patient is asymptomatic, and/or the event can be adequately treated with low molecular weight heparin-based therapy.

Patients with unresected primary tumors (or local recurrence) who develop Grade 3 and 4 venous thromboembolism may also receive anticoagulation and continue ramucirumab therapy (as detailed above), provided that the tumor does not confer an excessive bleeding risk, in the opinion of the patient’s physician.

Grade 3 and 4 arterial thromboembolic events, or any PE/DVT occurring or worsening during anticoagulant therapy, require permanent discontinuation of ramucirumab therapy. Any venous or arterial event leading to discontinuation of ramucirumab therapy will be considered serious and should be reported via the SAE mechanism.

### Bleeding (Hemorrhagic) Events

Serious hemorrhagic AEs have been reported from clinical studies investigating ramucirumab. Hemorrhagic complications are associated with some malignancies (ie, variceal bleeding from portal hypertension in hepatocellular carcinoma, lower GI hemorrhage from bowel metastases in ovarian carcinoma), although the rate of these complications varies considerably. As detailed in the IB, the incidences of hemorrhagic events to date, significant background incidence of bleeding in some malignancies, and use of concomitant antiplatelet therapy in some of the reported cases precludes any definitive association between bleeding and ramucirumab, although ongoing surveillance and identification (and exclusion) of patients with high bleeding risk remain essential.

Ramucirumab therapy should be discontinued in the event of any Grade 3 or 4 bleeding (hemorrhagic) event.

### Proteinuria

If, while on therapy, a patient has proteinuria ≥2+ per a dipstick or routine UA, a 24-hour urine collection should be conducted. If the protein level is 2g to 3g/24 hours, protocol therapy will be temporarily discontinued for up to 2 weeks and a 24-hour urine collection will be repeated. Ramucirumab will resume at a reduced dose level (6 mg/kg every other week) once the protein level returns to <2 g/24 hours. A second dose reduction (to 5 mg/kg every other week) is permitted if the protein level >2 g/24 hours recurs. The patient will have ramucirumab permanently discontinued if the protein level is >3 g/24 hours, if there is a third occurrence of

>2 g/24 hours, or if the protein level does not return to <2 g/24 hours within 2 weeks.

### Gastrointestinal Perforation

Patients with unresected (or recurrent) primary tumors, mesenteric or peritoneal disease who participate in this clinical trial may be at increased risk for gastrointestinal perforation and/or fistula due to the nature of their metastatic gastric cancer. All cases of perforation and fistula should be reported via the SAE mechanism.

Ramucirumab should be permanently discontinued in the event of a gastrointestinal perforation.

### Reversible Posterior Leukoencephalopathy Syndrome (RPLS)

Reversible posterior leukoencephalopathy syndrome is a clinical and radiologic syndrome typically consisting of reversible cortical neurological dysfunction and brain-imaging findings of subcortical edema involving the posterior circulation, particularly the occipital lobes (51). The symptoms of RPLS most often include generalized seizures, headache, delirium, and cortical blindness, although these may vary significantly and occasionally include focal neurological deficits (50,52,53). Magnetic resonance imaging represents the most reliable method for the diagnosis (52). Clinical symptoms and MRI abnormalities usually recover within days to weeks with proper management, although permanent neurologic dysfunction has been reported (50,51,52,54).

Reversible posterior leukoencephalopathy syndrome has been associated with multiple clinical conditions including hypertensive encephalopathy, eclampsia, and renal failure with hypertension as well as the use of both immunosuppressive and cytotoxic drugs (51,55). Reversible posterior leukoencephalopathy syndrome has been associated with the use of the anti- VEGF agent bevacizumab, as described in the prescribing information for this agent (54,56).

While the precise pathogenesis of RPLS has not been established, the pathophysiology may involve impaired cerebrovascular autoregulation leading to blood-brain barrier disruption and vasogenic edema (57). Although the pathogenesis of RPLS appears to be multifactorial, drug- induced endothelial damage and acute hypertension are frequently proposed causes of cerebrovascular dysfunction in RPLS (54).

Reversible posterior leukoencephalopathy syndrome should be identified and treated promptly in order to minimize potential for permanent neurological damage. Treatment encompasses careful control of BP, withdrawal of potentially causative medication, and administration of anticonvulsant agents to those experiencing seizures (56).

One SAE of RPLS has been reported in the double-blind, randomized, placebo-controlled Phase 3 colorectal cancer study CP12-0920 (I4T-MC-JVBB). The event was determined to be related to administration of all study drugs, including blinded investigational drug product. (The treatment assignment for this patient remains blinded.) Because hypertension is an identified risk for ramucirumab, physicians should control BP in accordance with established guidelines.

In addition, physicians should consider a diagnosis of RPLS in the setting of seizures, headache, nausea, delirium, visual changes, and/or other unexplained neurological symptoms, especially in combination with hypertension and MRI findings of hyperintensity on T2-weighted and fluid- attenuated inversion recovery images.

No cases of RPLS have been specifically associated with ramucirumab therapy to this time. If the diagnosis of RPLS is confirmed, ramucirumab must be permanently discontinued. All cases of RPLS must be reported via the SAE mechanism.

### Congestive Heart Failure (CHF)

An increased risk of CHF has been associated with some antiangiogenic therapeutic agents, particularly in patients with metastatic breast cancer previously/concomitantly treated with anthracyclines or with other risk factors for CHF, including prior radiotherapy to the left chest wall. Findings have ranged from asymptomatic declines in left ventricular (LV) ejection fraction to symptomatic CHF requiring treatment or hospitalization. Caution should be exercised when treating patients with clinically significant cardiovascular disease such as preexisting coronary artery disease or CHF. Patients with symptomatic CHF, unstable angina pectoris, or symptomatic or poorly controlled cardiac arrhythmia should not be enrolled in clinical trials with ramucirumab.

### Impaired Wound Healing

Impaired wound healing has been observed with some antiangiogenic agents. Ramucirumab will not be administered to patients who have undergone major surgery within 28 days prior to randomization or have undergone central venous access device placement within 7 days prior to randomization. Patients with postoperative and other nonhealing wound complications are excluded, as are patients for whom major surgical procedures are planned.

### Liver Failure

Any patient who experiences signs of hepatic encephalopathy or other serious signs of liver impairment, such as hepatorenal syndrome, must be permanently discontinued from ramucirumab therapy.

## Appropriateness of Measurements

Because there is no comparator group in this study, the evaluation of survival is not being undertaken. This study focuses on collecting those AEs likely to be of most consequence to patients: namely all SAEs, all Grade 3 or higher AEs, and all AESIs of any grade.

# DATA QUALITY ASSURANCE

This is a study designed to provide ramucirumab to appropriately selected patients prior to marketing authorization with a focus of collecting information about important safety findings experienced by patients while being treated on this study.

# . SAMPLE SIZE AND STATISTICAL METHODS

### Sample Size Calculation

A maximum of 61 patients was planned to be recruited to this single-arm phase II trial. The primary endpoint of this trial is overall response (OR=PR+CR). Wilke et al. (27) observed 16% of OR rate (ORR ) from a combination therapy called RAINBOW. We will not be interested in the experimental therapy of this trial if its ORR is P0=15% or lower and highly interested if its ORR is P1=30% or higher. A maximum of n=58 eligible patients (61 accounting for 5% of ineligibility) will be treated through the following 2-stage design.

Stage 1: n1=30 patients will be treated by the experimental therapy, and the trial will be stopped by rejecting the experimental therapy if 4 or fewer of them respond. Otherwise, we will proceed to Stage 2.

Stage 2: An additional 28 patients will be treated, and we will reject the study therapy if 13 or fewer of the cumulative 58 patients respond. Otherwise, the experimental therapy will be accepted for further investigation.

This 2-stage design has a one-sided alpha of 5% for P0=15% and a power of 86% for P1=30%.

**Statistical Analysis Plan**

To analyze the response rate according to molecular subtypes identified through ACRG/TCGA effort, we performed integrative genomic analysis to identify predictive markers (i.e. angiogensis signatures) for treatment response. All statistical analyses were performed using R3.4.0. Data was imported into R, plotted, computed, and *P*-values automatically added for significance levels, using “ggpubr” and visualized using “ggplot2”.

## Clinical Tests

### Laboratory Parameters

All laboratory tests will be performed at local labs and should be done to ensure patients meet all eligibility criteria and criteria to continue on the protocol:

Hematology Profile – Includes a complete blood count with differential and platelet count. Coagulation Profile – Includes INR, prothrombin time, and PTT.

Chemistry Profile – Includes creatinine, AST, ALT, alkaline phosphatase, total protein, and bilirubin.

NOTE: If pretreatment serum creatinine is >1.5 times the ULN, then a 24-hour urine collection must be performed, in order to calculate creatinine clearance and determine eligibility per Inclusion Criterion #11 (Section 7.1).

Urinalysis – Includes routine UA or dipstick measurements and, if clinically indicated, microscopic analysis. If dipstick or routine UA indicates pretreatment proteinuria ≥2+, a 24-hour urine collection (to assess protein) must be obtained.

Serum -HCG Pregnancy Test – Minimum sensitivity 25 IU/L or equivalent units of β-HCG, to be performed within 7 days prior to enrollment for WOCBP.

### Other Tests and Evaluations

Echocardiogram– Pretreatment only, to be obtained within 28 days prior to enrollment **only** for patients who have received prior anthracycline therapy. Subsequent evaluations are not required, but may be done at the discretion of the patient’s physician if warranted by dyspnea or other relevant symptoms.

## Criteria for Tumor Response Evaluation

Objective evaluation of disease control is highly recommended and will be conducted according to local practice.

## Symptomatic Deterioration (Clinical Progression)

As detailed in Section 7.3.1, deterioration in ECOG PS of ≥2 units compared to baseline is criteria for removal from ramucirumab therapy. Patients may also be removed from ramucirumab therapy if the attending physician determines any clinical deterioration is such that it is not in the patient’s best interest to continue on therapy (Section 7.3.1).

## Determination of Overall Response

No other evaluation of disease control will be requested. At discontinuation, information about planned subsequent therapy will be collected.

# INFORMED CONSENT, ETHICAL REVIEW AND REGULATORY CONSIDERATIONS

### Informed Consent

The patient’s physician is responsible for ensuring that informed consent is given by each patient or legal representative. This includes obtaining the appropriate signatures and dates on the ICF prior to the performance of any protocol procedures and prior to the administration of ramucirumab. Each patient or the patient’s legally acceptable representative will be required to read, agree to, and sign a current Institutional Review Board (IRB) approved informed consent form (ICF) prior to being enrolled and/or before any Study -related procedure is performed. In any countries where regulatory authorities do not utilize an IRB-approved ICF, each patient or the patient’s legally acceptable representative will be required to read, agree to, and sign a Sponsor-approved ICF.

### Ethical Review

Documentation of IRB approval of the protocol and the ICF must be provided to Lilly or its designee before the study may begin at that site. In any countries where regulatory authorities do not obtain IRB approval of the protocol and ICF, the signed Sponsor-approved ICF must be

provided to Lilly or its designee before the study may begin at that site.

### Regulatory Considerations

For countries requiring IRB approval per country-specific regulatory guidelines, the lead treating physician or designee will promptly submit the protocol to applicable IRB(s).

### Information for Treating Physicians

Physicians with a specialty in oncology who have participated in an externally sponsored clinical trial within the previous 24 months may participate in this study.

# REFERENCES

- 1. American Cancer Society. Cancer Facts and Figures 2012. Available at: [http://www.cancer.org/acs/groups/content/@epidemiologysurveilance/documents/document/acsp](http://www.cancer.org/acs/groups/content/%40epidemiologysurveilance/documents/document/acsp) c-031941.pdf. Accessed January 26, 2013.
  2. Wagner AD, Grothe W, Haerting J, Kleber G, Grothey A, Fleig W. Chemotherapy in advanced gastric cancer: A systematic review and meta-analysis based on aggregate data. *J Clin Oncol*. 2006;24:2903-2909.
  3. Cancer Research UK. Stomach cancer incidence statistics. Available at: <http://www.cancerresearchuk.org/cancer-info/cancerstats/types/stomach/incidence/uk-stomach-> cancer-incidence-statistics. Accessed April 5, 2013.
  4. Ferlay J, Shin HR, Bray F, Forman D, Mathers C, Parkin DM GLOBOCAN 2008 v1.2, Cancer Incidence and Mortality Worldwide: IARC CancerBase No. 10 [Internet]. Lyon, France: International Agency for Research on Cancer, 2010. Available at: [http://globocan.iarc.fr.](http://globocan.iarc.fr/) Accessed May 2011.
  5. Van Cutsem E, Moiseyenko VM, Tjulandin S, Majlis A, Constenla M, Boni C, et al. Phase III study of docetaxel and cisplatin plus fluorouracil compared with cisplatin and fluorouracil as first- line therapy for advanced gastric cancer: A report of the V325 Study Group. *J Clin Oncol*. 2006;24:4991-4997.
  6. Vanhoefer U, Rougier P, Wilke H, Ducreux M, Lacave AJ, Van Cutsem E, et al. Final results of a randomized phase III trial of sequential high-dose methotrexate, fluorouracil, and doxorubicin versus etoposide, leucovorin, and fluorouracil versus infusional fluorouracil and cisplatin in advanced gastric cancer. *J Clin Oncol*. 2000;18:2648-2657.
  7. Glimelius B, Ekstrom K, Hoffman K, Graf W, Sjoden PO, Haglund U, et al. Randomized comparison between chemotherapy plus best supportive care with best supportive care in advanced gastric cancer. *Ann Oncol*. 1997;8(2):163-168.
  8. Murad AM, Santiago FF, Petroianu A, Rocha PR, Rodrigues MA, Rausch M. Modified therapy with 5-fluorouracil, doxorubicin, and methotrexate in advanced gastric cancer. *Cancer*. 1993;72:37-41.
  9. Pyrhönen S, Kuitunen T, Nyandoto P, Kouri M. Randomised comparison of fluorouracil, epidoxorubicin and methotrexate (FEMTX) plus supportive care with supportive care alone in patients with non-resectable gastric cancer. *Br J Cancer*. 1995;71:587-591.
  10. Stein HJ, Feith M, Siewert JR. Cancer of the esophagogastric junction. *Surgical Oncology*. 2000;9:35-41.
  11. Swisher SG, Pisters PWT, Komaki R, Lahoti S, Ajani JA. Gastroesophageal junction

adenocarcinoma. *Current Treatment Options in Oncology*. 2000;1:387-398.

- 1. Shah MA, Ramanathan RK, Ilson DH, Levnor A, D’Adamo D, O’Reilly E, et al. Multicenter phase II study of irinotecan, cisplatin, and bevacizumab in patients with metastatic gastric or gastroesophageal junction adenocarcinoma. *J Clin Oncol*. 2006;24:5201-5206.
  2. Assersohn L, Brown G, Cunningham D, Ward C, Oates J, Waters JS, et al. Phase II study of irinotecan and 5-fluorouracil/leucovorin in patients with primary refractory or relapsed advanced esophageal and gastric carcinoma. *Ann Oncol*. 2004;15:64-69.
  3. Barone C, Basso M, Schinzari G, Pozzo C, Trigila N, D’Argento E, et al. Docetaxel and oxaliplatin combination in second-line treatment of patients with advanced gastric cancer. *Gastric Cancer*. 2007;10:104-111.
  4. Cunningham D, Starling N, Rao S, Iveson T, Nicolson M, Coxon F, et al. Capecitabine and oxaliplatin for advanced esophagogastric cancer. *N Engl J Med*. 2008;358:36-46.
  5. Kim ST, Kang WK, Kang JH, Park KW, Lee J, Lee S-H, et al. Salvage chemotherapy with irinotecan, 5-fluorouracil and leucovorin for taxane- and cisplatin-refractory, metastatic gastric cancer. *Br J Cancer*. 2005;92:1850-1854.
  6. Lee J-L, Ryu M-H, Chang HM, Kim T-W, Yook JH, Oh ST, et al. A phase II study of docetaxel as salvage chemotherapy in advanced gastric cancer after failure of fluoropyrimidine and platinum combination chemotherapy. *Cancer Chemother Pharmacol*. 2007; May 23 [e-pub ahead of print].
  7. Hartmann JT, Pintoffl JP, Al-Batran S-E, Quietzsch D, Meisinger I, Horger M, et al. Mitomycin C plus infusional 5-fluorouracil in platinum-refractory gastric adenocarcinoma: An extended multicenter phase II study. *Onkologie*. 2007;30:235-240.
  8. Giuliani F, Gebbia V, De Vita F, Maiello E, Di Bisceglie M, Catalano G, et al. Docetaxel as salvage therapy in advanced gastric cancer: A phase II study of the Gruppo Oncologico Italia Meridionale (GOIM). *Anticancer Research*. 2003;23:4219-4222.
  9. Giuliani F, Molica S, Maiello E, Battaglia C, Gebbia V, Di Bisceglie M, et al. Irinotecan (CPT-11) and mitomycin-C (MMC) as second-line therapy in advanced gastric cancer. *Am J Clin Oncol*. 2005;28:581-585.
  10. Thuss-Patience PC, Kretzschmar A, Bichev D, Deist T, Hinke A, Breithaupt K, et al. Survival advantage for irinotecan versus best supportive care as second-line chemotherapy in gastric cancer—a randomized phase III study of the Arbeitsgemeinschaft Internistische Onkologie (AIO). *Eur J Cancer*. 2011 47:2306-14.
  11. Kang JH, Lee SI, Lim DH, Park K-W, Oh SY, Kwon H-C, et al. Salvage Chemotherapy for Pretreated Gastric Cancer: A Randomized Phase III Trial Comparing Chemotherapy Plus Best Supportive Care With Best Supportive Care Alone. *J Clin Oncol*. 2012;30:1513-1518.
  12. Ohtsu A, Ajania JA, Bai Y-X, Bang Y-J, Chung H-C, Pan H-M, et al. Everolimus for previously treated advanced gastric cancer: results of the randomized, double-blind, phase III GRANITE-1 study. *J Clin Oncol.* 2013;31(31):3935-3943.
  13. Hironaka S, Ueda S, Yasui H, Nishima T, Tsuda M, Tsumura T, et al. Randomized, open- label, phase III study comparing irinotecan with paclitaxel in patients with advanced gastric cancer without severe peritoneal metastasis after failure of prior combination chemotherapy using a fluoropyrimidine plus platinum: WJOG 4007 trial*. J Clin Oncol.* 2013;31:4438-4444.
  14. Fuchs CS, Tomasek J, Yong CJ, Dumitru F, Passalacqua R, Goswami C, et al. Ramucirumab monotherapy for previously treated advanced gastric or gastro-oesophageal junction adenocarcinoma (REGARD): an international, randomised, multicentre, placebo-controlled, phase 3 trial. *Lancet.* 2014;383:31-39.
  15. Ford HER, Marshall A, Bridgewater JA, Janowitz T, Coxon FY, Wadsley J, et al. Docetaxel versus active symptom control for refractory oesophagogastric adenocarcinoma (COUGAR-02): an open-label, phase 3 randomised controlled trial. *Lancet Oncol.* 2014;15:78-86.
  16. Wilke H, Van Cutsem E, Oh SC, Bodoky G, Shimada Y, Hironaka S, et al. RAINBOW: a global, phase III, randomized, double-blind trial of ramucirumab plus paclitaxel versus placebo plus paclitaxel in the treatment of metastatic gastric or gastroesophageal junction (GEJ) and gastric adenocarcinoma following disease progression on first-line platinum- and fluoropyrimidine-containing combination therapy rainbow IMCL CLP12-0922 (I4T-JIE-JVBE). *J Clin Oncol, 2014 Gastrointestinal Cancers Symposium*. 2014; 32(suppl 3). Abstract LBA7.
  17. Folkman J. Angiogenesis in cancer, vascular, rheumatoid and other disease. *Nat Med*. 1995;1:27-31.
  18. Kerbel RS. Tumor angiogenesis: past, present and the near future. *Carcinogenesis*. 2000;21:505-515.
  19. Carmeliet P, Jain RK. Angiogenesis in cancer and other diseases. *Nature*. 2000;407:249-257.
  20. Klagsbrun M, D’Amore PA. Vascular endothelial growth factor and its receptors. *Cytokine Growth Factor Rev*. 1996;7:259-270.
  21. Liekens S, De Clercq E, Neyts J. Angiogenesis: regulators and clinical applications. *Biochem Pharmacol*. 2001;61:253-270.
  22. Ferrara N. The role of vascular endothelial growth factor in pathological angiogenesis. *Breast Cancer Res Treat*. 1995;36:127-137.
  23. Plate KH, Breier G, Millauer B, Ullrich A, Risau W. Up-regulation of vascular endothelial growth factor and its cognate receptors in a rat glioma model of tumor angiogenesis. *Cancer Res*. 1993;53:5822-5827.
  24. Witte L, Hicklin DJ, Zhu Z, Pytowski B, Kotanides H, Rockwell P, et al. Monoclonal

antibodies targeting the VEGF receptor-2 (Flk1/KDR) as an anti-angiogenic therapeutic strategy.

*Cancer Metastasis Rev*. 1998;17:155-161.

- 1. Zhu Z, Witte L. Inhibition of tumor growth and metastasis by targeting tumor-associated angiogenesis with antagonists to the receptors of vascular endothelial growth factor. *Invest New Drugs*. 1999;17:195-212.
  2. Hicklin D, Witte L, Zhu Z, Liao F, Wu Y, Li Y, et al. Monoclonal antibody strategies to block angiogenesis. *Drug Discovery Today*. 2001;6:517-528.
  3. Zhu Z, Bohlen P, Witte L. Clinical development of angiogenesis inhibitors to vascular endothelial growth factor and its receptors as cancer therapeutics. *Curr Cancer Drug Targets*. 2002;2:135-156.
  4. Lu D, Jimenez X, Zhang H, Bohlen P, Witte L, Zhu Z. Selection of high affinity human neutralizing antibodies to VEGFR2 from a large antibody phage display library for antiangiogenesis therapy. *Int J Cancer*. 2002;97:393-399.
  5. Lu D, Shen J, Vil MD, Zhang H, Jimenez X, Bohlen P, et al. Tailoring in vitro selection for a picomolar affinity human antibody directed against vascular endothelial growth factor receptor 2 for enhanced neutralizing activity. *J Biol Chem*. 2003;278:43496-43507.
  6. Mackey JR, Ramos-Vasquez M, Lipatov O, Kraznozhon D, Semiglazov V, Manikhas A, et al. Primary results of ROSE/TRIO-12, a randomized placebo controlled phase III trial evaluating the addition of ramucirumab to first-line docetaxel chemotherapy in metastatic breast cancer. Presented at: the 36th Annual San Antonio Breast Cancer Symposium held December 10-14, 2013, San Antonio, TX. Abstract S5-04.
  7. Feng CW, Wang LD, Jiao LH, Liu B, Zheng S, Xie XJ. Expression of p53, inducible nitric oxide synthase and vascular endothelial growth factor in gastric precancerous and cancerous lesions: Correlation with clinical features. *BMC Cancer*. 2002;2:8.
  8. Maeda K, Chung YS, Ogawa Y, Takatsuka S, Kang SM, Ogawa M, et al. Prognostic value of vascular endothelial growth factor expression in gastric carcinoma. *Cancer*. 1996;77:858-863.
  9. Yoshikawa T, Tsuburaya A, Kobayashi O, Sairenji M, Motohashi H, Yanoma S, et al. Plasma concentrations of VEGF and bFGF in patients with gastric carcinoma. *Cancer Letters*. 2000;153:7-12.
  10. Karayiannakis AJ, Syrigos KN, Polychronidis A, Zbar A, Kouraklis G, Simopoulos C, et al. Circulating VEGF levels in the serum of gastric cancer patients: Correlation with pathological variables, patient survival, and tumor surgery. *Ann Surg*. 2002;236:37-42.
  11. Jüttner S, Wissmann C, Jons T, Vieth M, Hertel J, Gretschel S, et al. Vascular endothelial growth factor-D and its receptor VEGFR-3: Two novel independent prognostic markers in gastric adenocarcinoma. *J Clin Oncol*. 2006;24:228-240.
  12. Jung YD, Mansfield PF, Akagi M, Takeda A, Liu W, Bucana CD, et al. Effects of combination anti-vascular endothelial growth factor receptor and anti-epidermal growth factor receptor therapies on the growth of gastric cancer in a nude mouse model. *Eur J Cancer*. 2002;38:1133- 1140.
  13. Eisenhauer EA, Therasse P, Bogaert J, Schwartz LH, Sargent D, Ford R, et al. New response evaluation criteria in solid tumours: revised RECIST guideline (version 1.1). *Eur J Cancer*. 2009;45(2) 228-247.
  14. The Criteria Committee of the New York Heart Association. Nomenclature and Criteria for Diagnosis of Diseases of the Heart and Great Vessels, Ninth Edition. Boston, MA: Little, Brown & Co; 1994.
  15. Avastin® (bevacizumab) [package insert]. San Francisco, CA. Genentech, Inc. October 2006.
  16. Hinchey J, Chaves C, Appignani B, Breen J, Pao L, Wang A, et al. A reversible posterior leukoencephalopathy syndrome. *N Engl J Med*. 1996;334:494–500.
  17. Garg RK. Posterior leukoencephalopathy syndrome. *Postgrad Med J*. 2001;77:24–28.
  18. Lee VH, Wijdicks EFM, Manno EM, Rabinstein AA. Clinical spectrum of reversible posterior leukoencephalopathy syndrome. *Arch Neurol*. 2008;65:205–210.
  19. Tajima Y, Isonishi K, Kashiwaba T, Tashiro K. Two similar cases of encephalopathy, possibly a reversible posterior leukoencephalopathy syndrome: serial findings of magnetic resonance imaging, SPECT, and angiography. *Intern Med*. 1999;38:54–58.
  20. Marinella MA, Markert RJ. Reversible posterior leucoencephalopathy syndrome associated with anticancer drugs. *Intern Med J*. 2009;39:826–834.
  21. Stott VL, Hurrell MA, Anderson TJ. Reversible posterior leukoencephalopathy syndrome: a misnomer reviewed. *Intern Med J*. 2005;35:83–90.
  22. Schwartz RB. A reversible posterior leukoencephalopathy syndrome. *N Engl J Med*. 1996;334:174.

# APPENDIX 1

## 14.1. Post-Enrollment Evaluations: Frequency of Post-Enrollment Evaluations

| **Procedure** | **Required Pre- Treatment**  **Evaluations^a^** | **Treatment Cycles^b^** | | **End of Therapy^c^** |
| --- | --- | --- | --- | --- |
|  |  | **q 2 w every cycle** | **q 6 w**  **every 3 cycles** |  |
| **Eligibility Assessments** |  |  |  |  |
| Informed Consent  Medical/Oncologic History (including record of preexisting toxicity, if any) | X X  Xd |  |  |  |
| Pregnancy Test Electrocardiogram ECOG PS  Echocardiogram/MUGA | Xe  X  Xm |  | Xi | X |
| **Safety Assessments**  Physical Exam, Height, Weight  Vital Signs (blood pressure, pulse rate, respiration rate, temperature)^g^ Toxicity Assessments/Adverse Events | X X  X | X X  Xj |  | X  Xj |
| **Laboratory Tests** | X X X X (X^l^) |  |  |  |
| Hematology Profile Coagulation Profile Chemistry Profile Urinalysis  24-Hour Urine Collection |  | Xh X | X X | X X X X |
| **Disease Control Assessments**  Imaging (CT/MRI) Tumor Assessments | Xf Xf |  | X X | X  Xj |
| **Clinical Drug Supplies**  Administer ramucirumab |  | X k |  |  |
| **Biopsy specimensn** | X |  |  | At progression |
| **Blood for genomics (10 ml ACD)** | X |  |  |  |

**Post-Enrollment Evaluations for Protocol I4T-MC-JVCP Frequency of Post-Enrollment Evaluations**

Abbreviations: CT = computed tomography; ECOG PS = Eastern Cooperative Oncology Group performance status; MRI = magnetic resonance imaging; MUGA = multiple gated acquisition scan; q 2 w = every 2 weeks; q 6 w = every 6 weeks.

a. Pretreatment evaluations will be performed within 28 days prior to enrollment, unless otherwise specified.

b. Treatment cycle defined herein as 2 weeks; however, there will be no treatment interruption or break between cycles. Patients will continue to undergo treatment according to program protocol until there is evidence of disease progression, toxicity requiring cessation, withdrawal of consent, or until other withdrawal criteria are met.

c. End of therapy evaluations will be performed at the discontinuation of therapy; patients should be followed for at least 30 days after last dose of protocol therapy to ensure sufficient safety monitoring and any AEs reported unless another therapy has been commenced **and** the AE is thought related to post-program therapy. Written informed consent must be obtained prior to any program-specific pretreatment evaluations and before patient enrollment.

d. WOCBP must have a negative serum pregnancy test (minimum sensitivity 25 IU/L or equivalent units of β-HCG) within 7 days prior to enrollment.

e. To be obtained within 28 days prior to enrollment only for patients with prior anthracycline therapy. Subsequent evaluations are not required, but may be done at the discretion of the patient’s physician if warranted by dyspnea or other relevant symptoms.

f. Baseline imaging to determine the extent of disease and identify metastases, and tumor assessments should be performed within 14 days prior to enrollment, and every 6 weeks (±3 days) until documented progression for patients with disease status of complete response (CR), partial response (PR), or stable disease (SD), and/or for patients who have discontinued program therapy due to toxicity or reasons other than progressive disease.

g. Including temperature, pulse rate, respiration rate, and blood pressure; to be obtained before, during, and at the completion of each infusion.

h. In situations where hemoglobin is less than 9 g/dL (5.58 mmol/L) and there are signs or symptoms of bleeding, a hematology profile should be performed weekly until hemoglobin is ≥9 g/dL (5.58 mmol/L) and any bleeding-related signs or symptoms have resolved or been adequately investigated.

i. Every 6 weeks or in accordance with local regulations, whichever is of shorter duration.

j. All SAEs considered at least possibly related to ramucirumab will be followed until resolution, stabilization, return to baseline, or until deemedirreversible.

k. It is recognized that in the course of clinical cancer care, it is not always possible to schedule therapeutic infusions precisely 2 weeks following a prior infusion (because of holidays, travel difficulties, or other circumstances). Accordingly, infusions administered within 3 days before or after the planned 2-week time point will be considered acceptable. Infusions outside this window are strongly discouraged.

l. If pretreatment serum creatinine is >1.5 times the ULN, then a 24-hour urine collection must be performed, in order to calculate creatinine clearance and determineeligibility.

m. Should be completed if patients had received prior anthracycline therapy.

n. Fresh tissues will be obtained: preferably from endoscopy. 4 to 8 pieces from tumor, 4 pieces of normal mucosa will be collected for DNA/RNA sequencing. Baseline and at progression. For ascites, 1 liter of ascites will be collected at baseline and progression and malignant cells will be isolated. For pleural effusion 500 cc to 1 liter will be collected. Any combination of endoscopic biopsy and ascites/pleural/CSF etc will be allowed in the protocol.

Post-Enrollment Evaluations for Protocol I4T-MC-JVCP Frequency of Post-

Enrollment Evaluations

c End of therapy evaluations will be performed at the discontinuation of therapy; patients should be followed for at least 30 days after last dose of protocol therapy to ensure sufficient safety monitoring and any AEs reported unless another therapy has been commenced **and** the AE is thought related to post-program therapy. Written informed consent must be obtained prior to any program-specific pretreatment evaluations and before patient enrollment.

# Appendix 2

Lab manual (under preparation for MFDS approval).
