# Supplementary material for: Comprehensive molecular characterization of gastric cancer patients from phase II second-line ramucirumab plus paclitaxel therapy trial
Source: Genome Med. 2021 Jan 25;13:11. doi: 10.1186/s13073-021-00826-w (PMC7836461; doi:10.1186/s13073-021-00826-w)
Supplement: Supplementary file 3 — Additional file 3: Figure S1. Immune cell composition of gastric cancer patients based on clinical response to ramucirumab. Figure S2. TCGA gastric cancer patient survival analysis based on ramucirumab-resistant signature activities. Figure S3. Pathway enrichment analysis between ramucirumab responder and non-responder patients. Figure S4. A heatmap of angiogenesis-associated pathway analysis. Figure S5. Elastic net-regression model-based analysis. [file 13073_2021_826_MOESM3_ESM.pptx]

## Slide 1
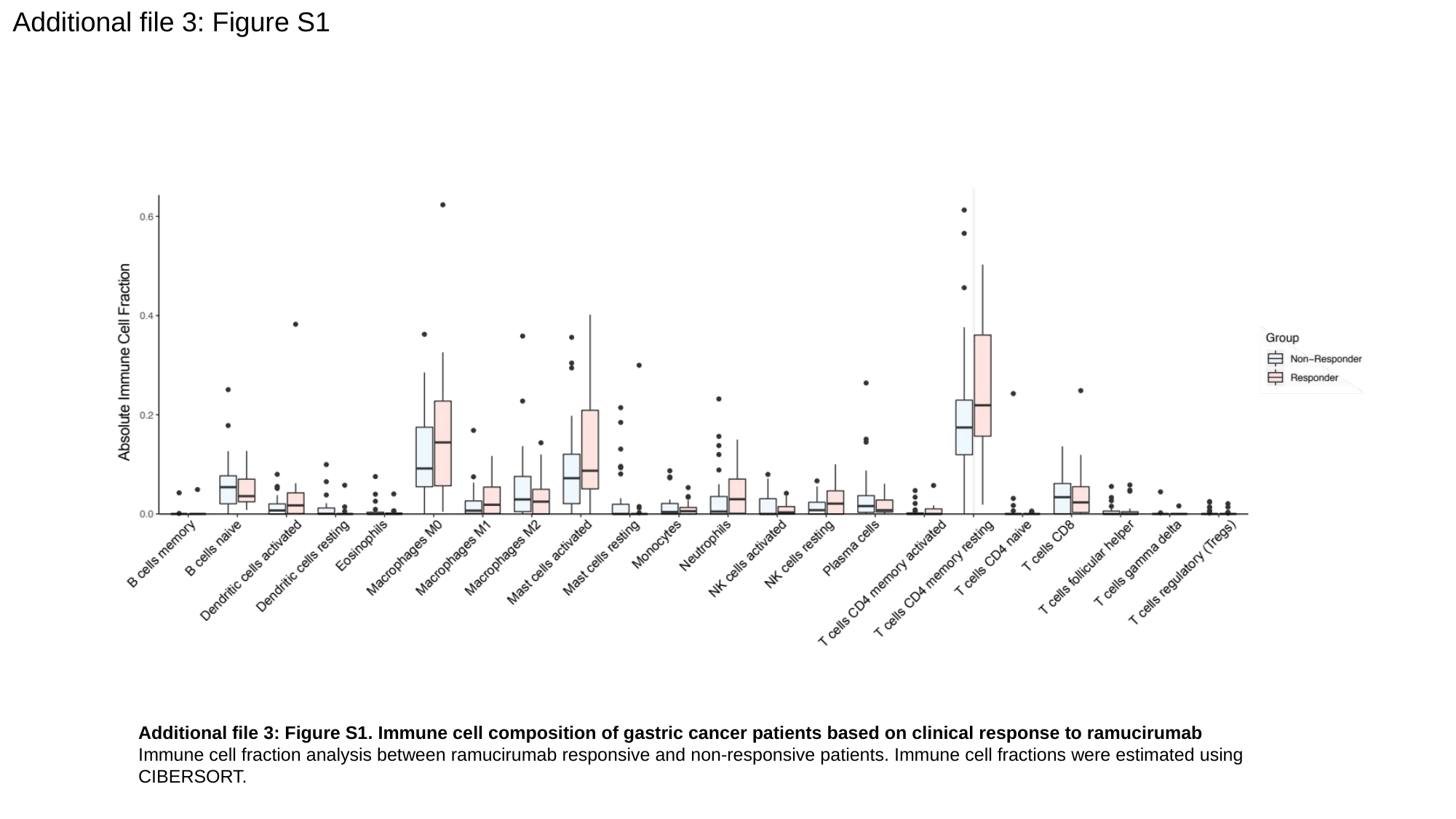

Additional file 3: Figure S1
Additional file 3: Figure S1. Immune cell composition of gastric cancer patients based on clinical response to ramucirumab
Immune cell fraction analysis between ramucirumab responsive and non-responsive patients. Immune cell fractions were estimated using CIBERSORT.

## Slide 2
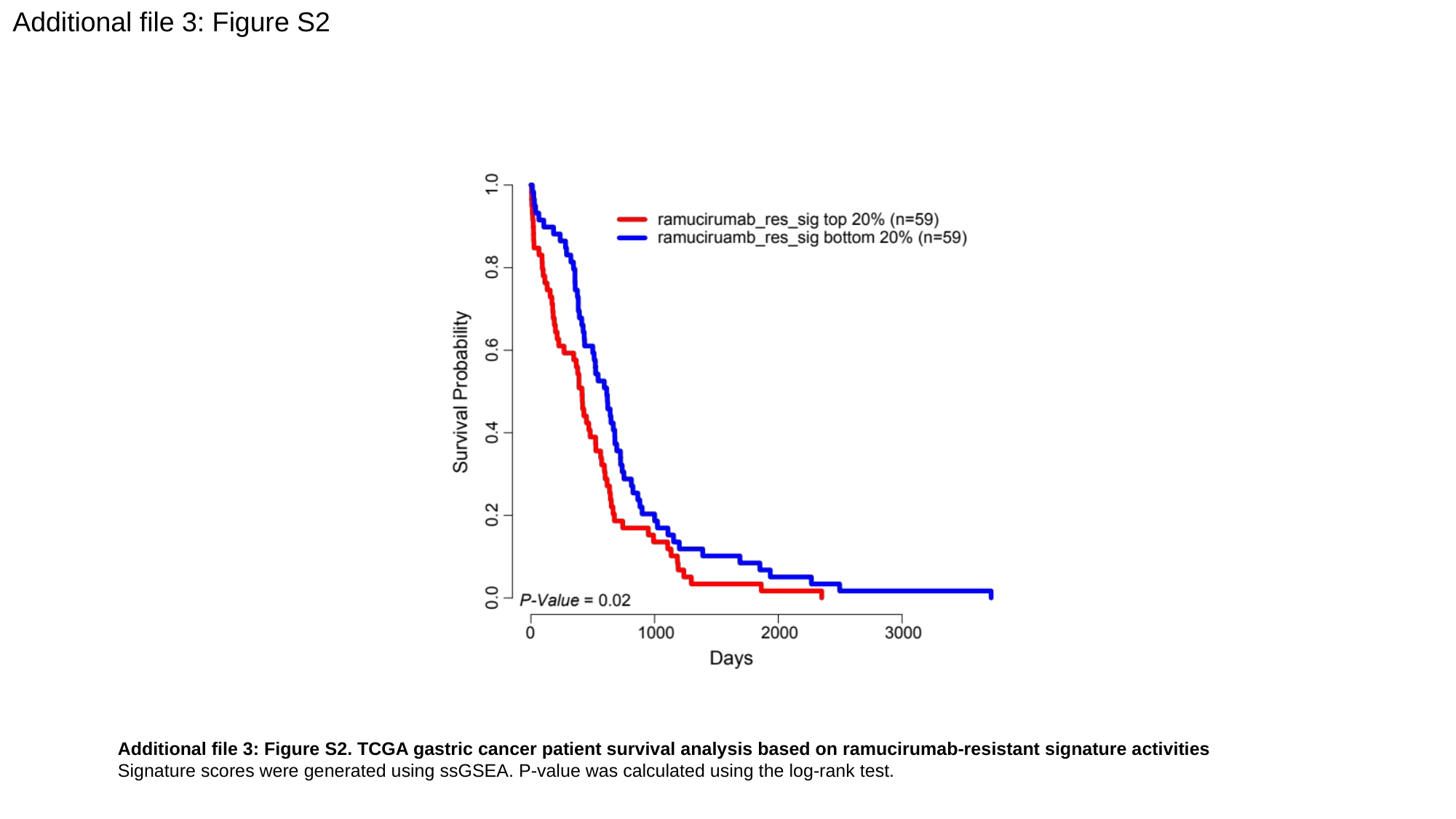

Additional file 3: Figure S2
Additional file 3: Figure S2. TCGA gastric cancer patient survival analysis based on ramucirumab-resistant signature activities
Signature scores were generated using ssGSEA. P-value was calculated using the log-rank test.

## Slide 3
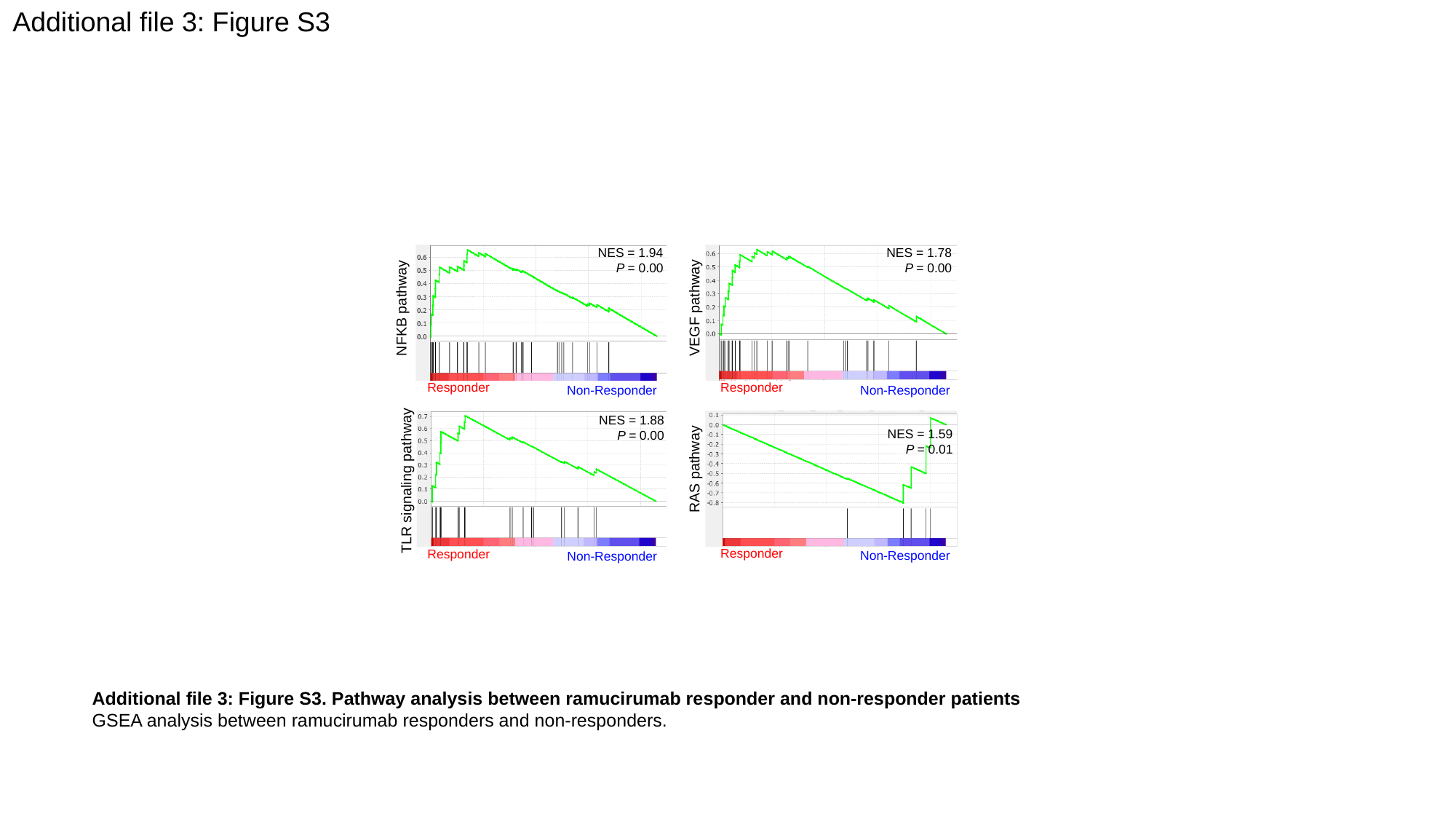

Additional file 3: Figure S3
NES = 1.78
P = 0.00
NES = 1.94
P = 0.00
NFKB pathway
VEGF pathway
Responder
Responder
Non-Responder
Non-Responder
NES = 1.88
P = 0.00
NES = 1.59
P = 0.01
RAS pathway
TLR signaling pathway
Responder
Responder
Non-Responder
Non-Responder
Additional file 3: Figure S3. Pathway analysis between ramucirumab responder and non-responder patients
GSEA analysis between ramucirumab responders and non-responders.

## Slide 4
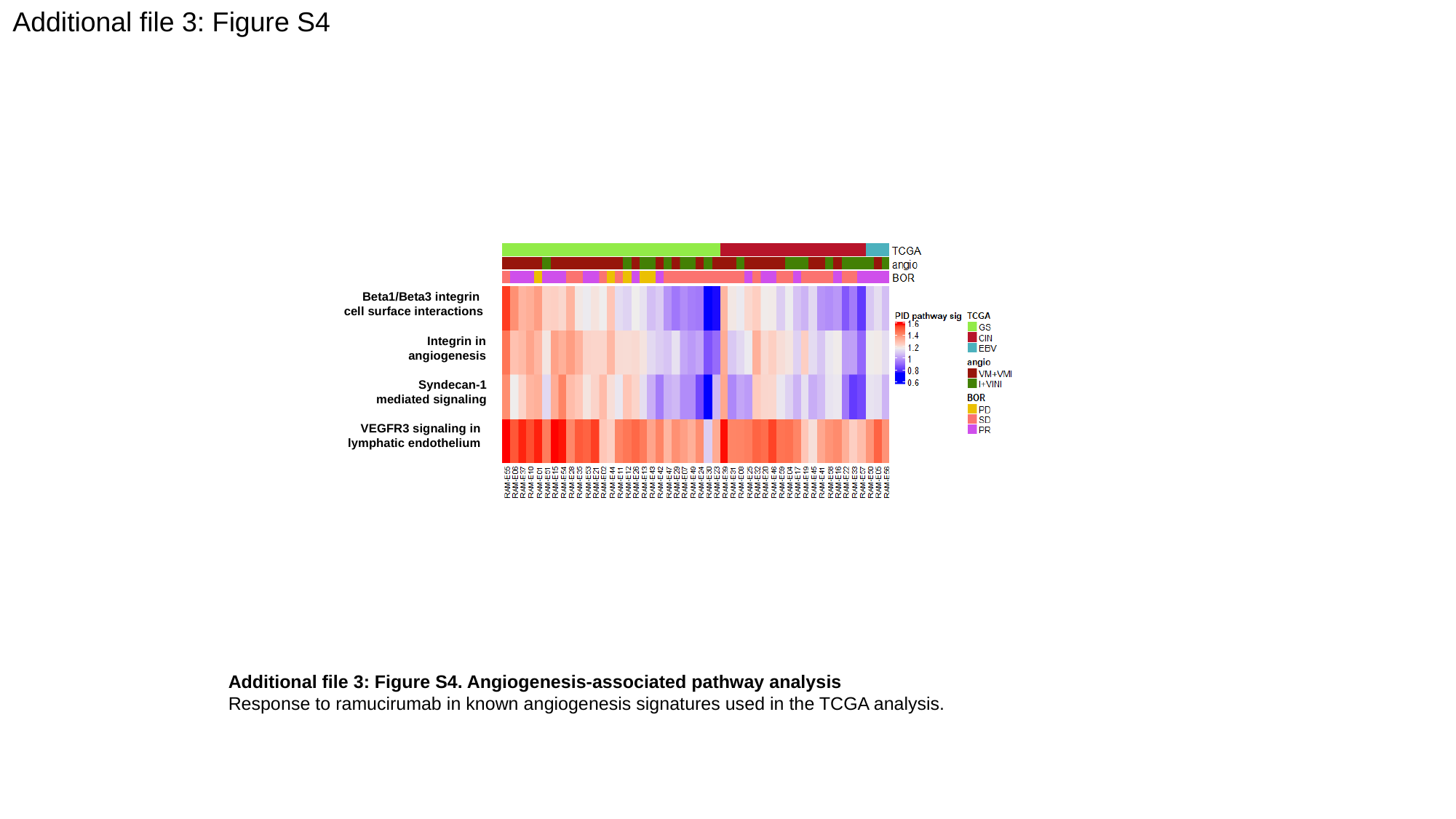

Additional file 3: Figure S4
Beta1/Beta3 integrin
cell surface interactions
Integrin in
angiogenesis
Syndecan-1
mediated signaling
VEGFR3 signaling in
lymphatic endothelium
Additional file 3: Figure S4. Angiogenesis-associated pathway analysis
Response to ramucirumab in known angiogenesis signatures used in the TCGA analysis.

## Slide 5
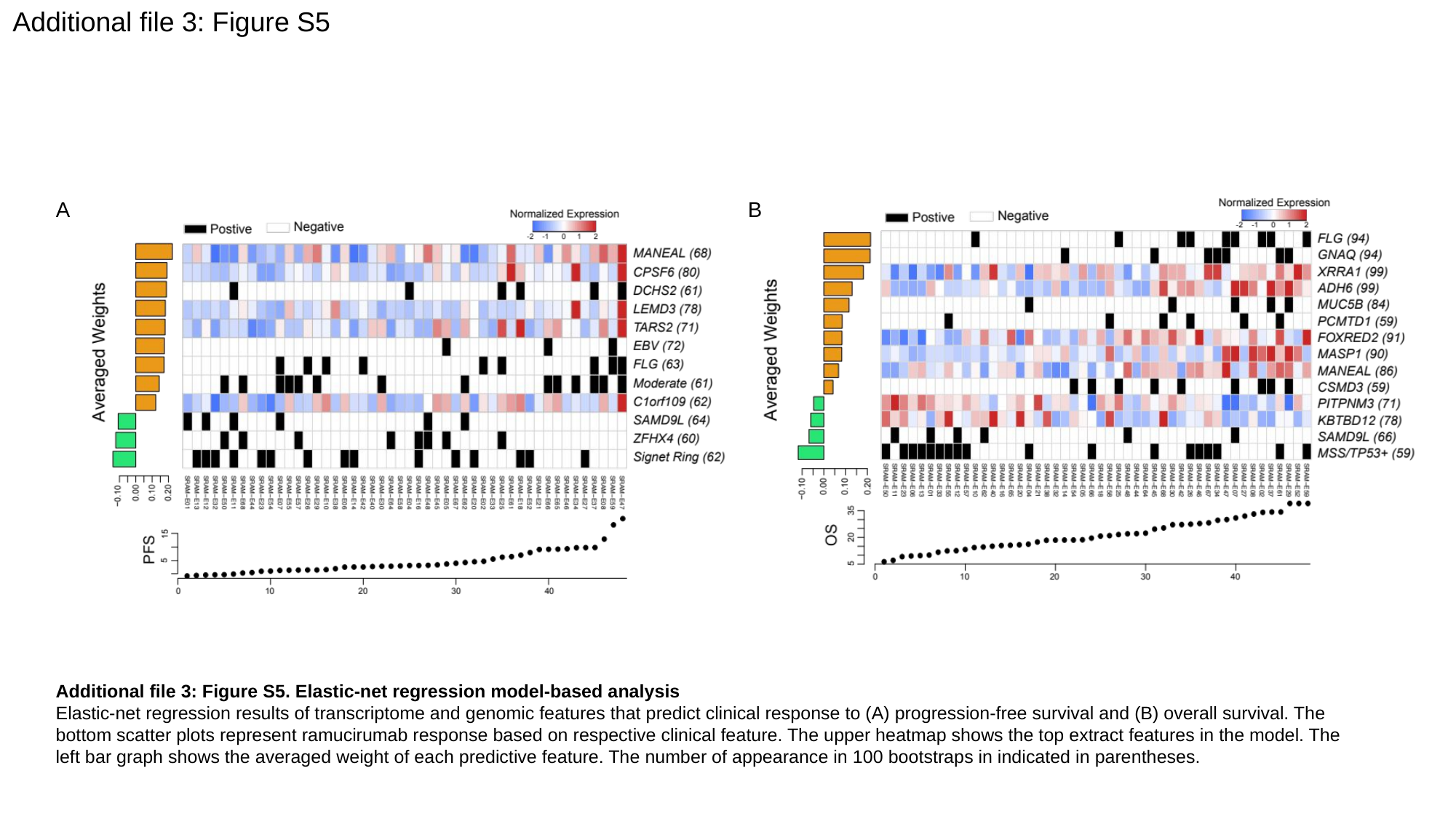

Additional file 3: Figure S5
A
B
Additional file 3: Figure S5. Elastic-net regression model-based analysis
Elastic-net regression results of transcriptome and genomic features that predict clinical response to (A) progression-free survival and (B) overall survival. The bottom scatter plots represent ramucirumab response based on respective clinical feature. The upper heatmap shows the top extract features in the model. The left bar graph shows the averaged weight of each predictive feature. The number of appearance in 100 bootstraps in indicated in parentheses.
